# In Silico Evaluation of Mechanobiological Parameters Under Variable Flow in Three-Dimensional Microfluidic Platforms Supporting Future Cell Migration Studies

**DOI:** 10.3390/biomedicines14091879

**Published:** 2026-08-23

**Authors:** Juan M. Munoz, Nicole M. E. Valle, Camilla M. Liu, Arielly H. Alves, Giovana F. Pileggi, Javier B. Mamani, Mariana F. Costa, Keithy F. da Silva, Marta C. S. Galanciak, Gabriel M. Rosário, Marcelo N. P. Carreño, Mariana P. Nucci, Alejandro Sosnik, Lionel F. Gamarra

**Affiliations:** 1 Hospital Israelita Albert Einstein, São Paulo 05652-000, Brazil; juanmunoz@usp.br (J.M.M.); nicolemev@gmail.com (N.M.E.V.); camillamanxiuliu123@gmail.com (C.M.L.); ariellydahora1997@gmail.com (A.H.A.); giovana.pileggi@gmail.com (G.F.P.); javierbm@einstein.br (J.B.M.); marianafecosta@hotmail.com (M.F.C.); keithyflx@gmail.com (K.F.d.S.); marta.caetano.2004@gmail.com (M.C.S.G.); gabrielmrosario16@gmail.com (G.M.R.); 2 Departamento de Engenharia de Sistema Eletrônicos, Escola Politécnica, Universidade de São Paulo, São Paulo 05508-010, SP, Brazil; mnpcarreno@usp.br; 3 LIM44—Laboratório de Investigação Médica em Neurorradiologia, Hospital das Clínicas, Faculdade de Medicina, Universidade de São Paulo, São Paulo 04453-030, SP, Brazil; mariana.nucci@hc.fm.usp.br; 4 Laboratory of Pharmaceutical Nanomaterials Science, Faculty of Materials Science and Engineering, Technion-Israel Institute of Technology, Haifa 3200003, Israel; sosnik@technion.ac.il

**Keywords:** cell migration, mechanobiology, microfluidics, wall shear stress, computational fluid dynamics, organ-on-a-chip

## Abstract

**Background:** Cell migration is a biological process influenced by biochemical signals and mechanical stimuli from the microenvironment. In this context, the accurate characterization of the mechanical microenvironment generated within microfluidic platforms represents an essential step for the design and interpretation of cell migration studies. Understanding how hydrodynamic forces influence the mechanical microenvironment experienced by cells remains a challenge, especially in confined and biomimetic systems. **Methods:** In this study, a three-dimensional microfluidic device was developed in silico to characterize the effects of flow variation on mechanofluidic parameters and to provide a quantitative basis for designing future cell-migration experiments. Computational fluid dynamics simulations were performed to characterize the velocity, pressure, and wall shear stress (WSS) distributions under different inlet flow rates (0.5, 1, and 5 µL/min) and three distinct inlet/outlet configurations within the same three-dimensional geometry. Rigid hemispherical probe structures were incorporated into the model to quantify the local shear stress acting on cell-sized surfaces. **Results:** The results demonstrated a direct and linear relationship between the applied flow rate and the WSS, modulated by the channel geometry and the inlet and outlet configuration. Regions near micropores and lateral channels showed high WSS values, while central regions experienced less mechanical stimulation, depending on flow conditions. Comparison with WSS values and ranges associated with cellular responses reported in the literature indicated that certain operational configurations generated mechanical conditions comparable to those previously investigated in cell-based studies, including cell migration applications. **Conclusions:** Overall, the study highlights the importance of controlling flow conditions in microfluidic platforms and provides a quantitative basis for the development and optimization of three-dimensional microfluidic devices intended for designing future cell-migration experiments. The systematic comparison of three inlet/outlet configurations across three flow rates within the same three-dimensional geometry provides a comparative framework for identifying configuration-dependent changes in the local mechanofluidic environment, supporting the selection of operational conditions for future mechanobiological and cell-migration studies.

## 1. Introduction

Cell migration is an essential biological process involved in embryonic development [1], immune responses [2], tumor metastasis [3], and other cellular functions [4]. This process is regulated by the integration of biochemical signals and physical stimuli from the microenvironment, which influence cytoskeletal organization and the formation of cellular adhesions [5,6], three-dimensional cell migration [7], directed cell migration [8], and the generation of traction forces [9].

Different migration mechanisms reflect cellular adaptations to these conditions, including actin-polymerization- and cell–ECM-adhesion-dependent migration [9], hybrid amoeboid–mesenchymal migration [10], the osmotic motor model [11], and the nuclear piston model [9]. Given this diversity, cell migration depends not only on biochemical signals but also on how cells sense and respond to physical forces within the microenvironment [12]. The conversion of these physical stimuli into biological responses, known as mechanotransduction, is a central aspect of mechanobiology [13]. Cells can sense mechanical forces and geometric features of their microenvironment, which can modulate their migratory behavior [14]. In this context, shear stress, hydrodynamic pressure, mechanical stretching, and spatial confinement can influence cellular polarization, directionality, adhesion, and migration.

Conventional two-dimensional (2D) cell cultures under static conditions are insufficient to faithfully replicate these physiological conditions, as they do not reproduce the three-dimensional microenvironmental cues present in native tissues [15]. In addition, matrix stiffness and spatial confinement can independently influence tumor-cell migration [16]. Static 2D cultures also lack the dynamic flow conditions characteristic of microfluidic environments. In this context, microfluidic devices emerge as advanced tools, with the potential to provide precise control of the physicochemical parameters of the microenvironment [17], and to support a wide range of physiological and biomedical applications, such as cell migration [18], drug delivery [19], nanoparticle toxicity [20], and cell viability [21]. However, the biological relevance of these platforms depends not only on the ability to maintain cells in culture, but also on the generation of mechanical conditions compatible with the maintenance of cellular viability. Since hydraulic resistance depends on channel geometry and dimensions [22,23], velocity, pressure, and shear stress distributions are highly sensitive to geometric variations and operational conditions. Mechanobiological studies based on microfluidic devices have demonstrated that even small variations in the shear stress field can modulate cell signaling pathways, including those involving Rho GTPases and integrins, as well as cytoskeletal organization and cell migration patterns [24]. Cells exposed to flows of different intensities also exhibit distinct orientations [12]. While flow velocity influences the supply of nutrients and oxygen to cells, among other biological outcomes [25], pressure gradients contribute to the regulation of cellular homeostasis [24,26], and WSS can modulate cell physiological responses [27,28]. The biological microenvironment arises from the interplay of various mechanofluidic factors [29], which makes a careful evaluation of each parameter essential for accurately estimating which one best corresponds to the biological response observed in experimental assays. These results indicate that uncontrolled mechanical variations within microfluidic devices can directly interfere with cell behavior, producing responses that may not exclusively reflect the biological phenomenon under investigation [24]. Therefore, understanding how device geometry and flow conditions shape the mechanobiological microenvironment is essential before their application in biological assays.

An important approach to better understand these mechanobiological parameters involves integrating computational modeling and experimental analysis through the use of computational fluid dynamics (CFD). This approach enables the simulation of the spatial distribution of velocity, pressure, and shear stress gradients [30], enabling the precise quantification of forces acting on cell-sized structures at the micrometer scale. CFD analysis also enables the assessment of how microchannel geometry influences the spatial distribution of velocity, pressure, and WSS [31]. In addition to optimizing microchannel design, this strategy provides a quantitative understanding of the mechanical microenvironment under different flow regimes and experimental protocols, which are fundamental for the application of microfluidic devices. Previous studies have reported distinct cellular responses to different shear stress ranges in endothelial cells, stem cells, cancer cells, fibroblasts, and osteoblasts under various flow regimes [32,33]. These cell-type- and flow-dependent responses provide relevant experimental information for defining mechanical conditions in modeling and CFD analyses.

Previous computational studies have characterized velocity, pressure, and WSS in microfluidic systems using different channel geometries and flow conditions [30,31,32,33], while organ-on-a-chip studies have investigated biologically relevant microfluidic architectures and tissue-specific environments [34,35]. Other studies have combined computational or experimental microfluidic approaches with the investigation of flow-induced cellular responses and cell migration [36,37,38]. These studies have therefore addressed either geometry-dependent hydrodynamics or biological responses under specific experimental configurations, but have not systematically isolated the effect of changing inlet/outlet configurations while maintaining the same three-dimensional five-channel geometry. In addition, local WSS quantification on wall-attached cell-sized hemispherical probes has not been evaluated together with the global hydrodynamic field across these multiple operational configurations. This unresolved point defines the specific contribution addressed in the present study.

Despite these advances, limited information is available on the combined influence of different inlet/outlet configurations on the global hydrodynamic field and local WSS at wall-attached cell-sized structures within the same three-dimensional microfluidic geometry. To address this gap, the present study had three objectives: (i) to computationally characterize the velocity, pressure, and WSS fields generated within a three-dimensional five-channel microfluidic device; (ii) to compare three inlet/outlet configurations at three prescribed flow rates while maintaining the device geometry unchanged; and (iii) to identify spatially distinct mechanical conditions that may be considered when designing future biological experiments. The study is limited to the computational characterization of the device-generated mechanical environment and does not directly measure, validate, or predict cellular migration behavior. Rigid wall-attached hemispherical structures were used as geometric probes for local WSS quantification and were not intended to reproduce the deformability, adhesion, traction generation, or biological behavior of living cells.

The specific contribution of this study is the systematic comparison of multiple inlet/outlet configurations within the same fixed three-dimensional five-channel geometry, combined with the quantification of local WSS on wall-attached cell-sized probe structures at predefined positions. This approach enables the effects of operational flow configuration on both the global hydrodynamic field and local probe-level WSS to be evaluated while maintaining the device geometry unchanged. By combining computational fluid dynamics (CFD) simulations with a mechanofluidic interpretation of the results, and contextualizing the predicted mechanical environment within existing mechanobiological evidence, this work seeks to establish a quantitative framework linking device geometry and operational conditions to the resulting mechanical environment, thereby supporting the rational design, operation, and interpretation of microfluidic platforms for mechanobiology and organ-on-a-chip applications, including cell migration studies.

## 2. Materials and Methods

### 2.1. Device Design and Characterization of Flow Simulation

**Figure 1:**
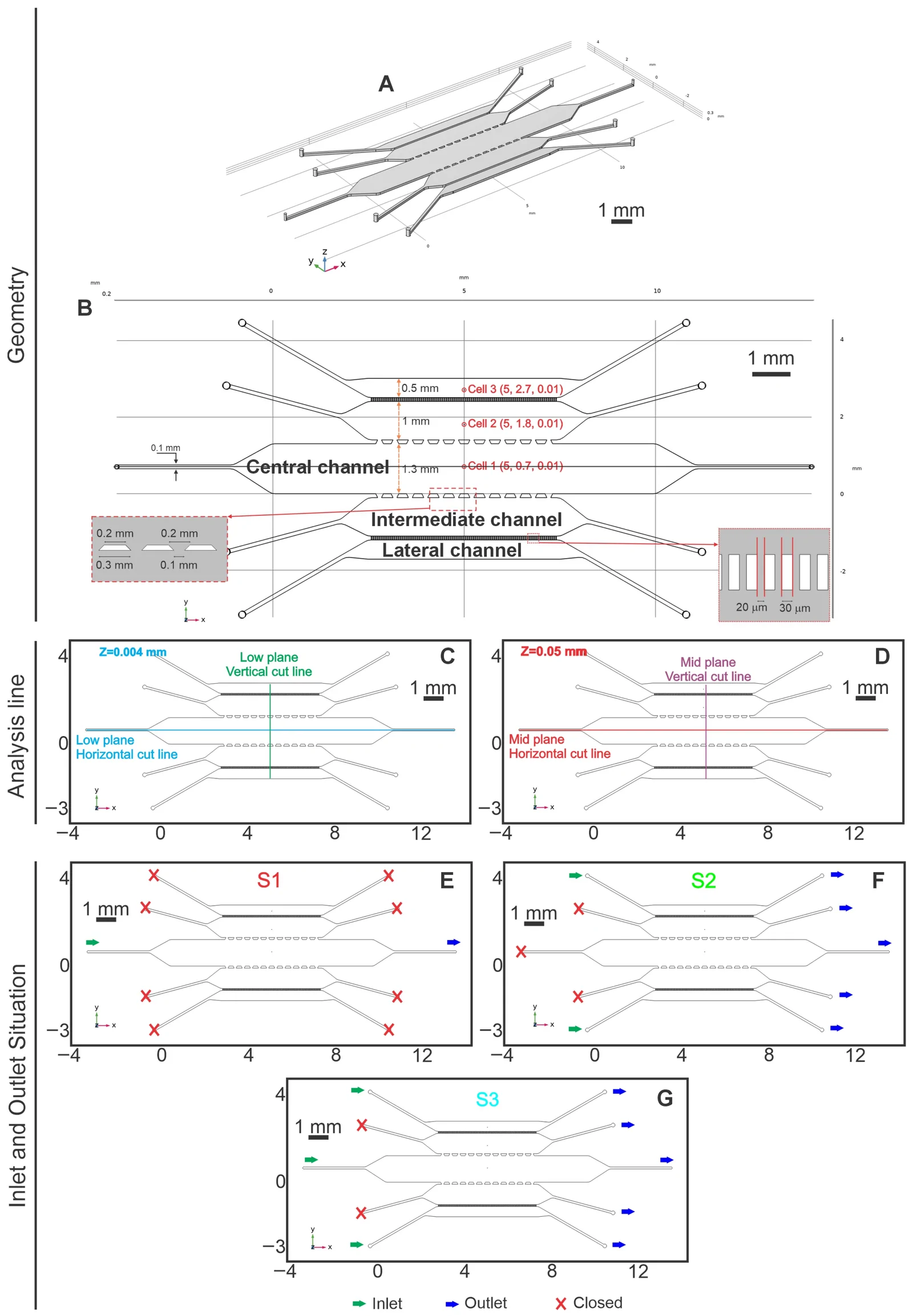
Complete geometry of the symmetric five-channel microfluidic device. (**A**) Three-dimensional view. (**B**) XY view of the microfluidic device showing the main dimensions of the channels and micropores, with the coordinates of Cells 1, 2, and 3 indicated in red. The micropores are 20 μm wide with 30 μm spacing, and the trapezoids have a minor base of 0.2 mm and a major base of 0.3 mm, as shown in the expanded view. The locations of the analyzed planes are also indicated. (**C**) Two-dimensional view of the low plane (z = 0.004 mm), with horizontal (blue line, y = 0.7) and vertical (green line, x = 5) cut lines used to obtain numerical values. (**D**) Two-dimensional view of the mid plane (z = 0.05 mm), with horizontal (red line, y = 0.7) and vertical (purple line, x = 5) cut lines used to obtain numerical values. (**E**–**G**) Inlet/outlet configurations for situations (S1–S3), respectively. In S1, the inlet and outlet are located in the central channel, while all other ports are closed. In S2, the inlets are positioned in the lateral channels and the outlets are open in all channels, while the central and intermediate inlets remain closed. In S3, the inlets are located in both the lateral and central channels, with outlets open in all channels, while the intermediate inlets remain closed. Green arrows indicate inlets, blue arrows indicate outlets, and red crosses indicate closed ports.

The design of the microfluidic device was conceived based on previous studies on organ-on-a-chip platforms [34,35], with the purpose of investigating tumor microenvironment dynamics. The developed chip has overall dimensions of 20 mm in length × 7.5 mm in width × 0.1 mm in height and incorporates five parallel channels interconnected by 0.008 mm height microporous partitions with distinct structural characteristics, as illustrated in Figure 1A. The main purpose of its construction is to evaluate the distribution of flow velocity, pressure, and WSS in the entire microfluidic device.

Figure 1B illustrates the device in the XY plane, with its primary dimensions delineated on the image grid. The widths of the outer, intermediate, and central channels are 0.5 mm, 1.0 mm, and 1.3 mm, respectively. The trapezoidal structures separating the central and intermediate channels feature a 0.3 mm major base, a 0.2 mm minor base, and a height of 0.1 mm, with a 0.1 mm gap between adjacent major bases. Micropores separating the intermediate and lateral channels are 20 µm wide with 30 µm spacing. Furthermore, the coordinates for three probe points, located at the apex of three cell-sized hemispherical probe structures integrated into the geometry, are indicated. Three idealized cell-sized probes are integrated into the device geometry, with their locations defined by the apex coordinates of each probe structure: Cell 1 at (5, 0.7, 0.01), Cell 2 at (5, 1.8, 0.01), and Cell 3 at (5, 2.7, 0.01).

For the analysis of hydrodynamic parameters within the device, two cut planes were defined at positions z = 0.05 mm (mid plane) and z = 0.004 mm (low plane), the latter corresponding to half the micropore height, to obtain the values associated with the theoretical maximum velocity intensity within the micropores. These planes were used to evaluate the spatial distributions of velocity, pressure, and WSS.

To obtain the nominal values of the main physical parameters (velocity, pressure, and WSS), two cut lines were established in each cut plane described above. The vertical lines were defined at a constant x-coordinate of 5 mm (shown in green line for low plane and purple for mid plane), while the horizontal lines were defined at y = 0.7 mm (shown in blue line for low plane and red line for mid plane), as shown in Figure 1C,D. The boundary conditions related to the flow inlets, outlets, and closed channels are detailed in Figure 1E–G, for each experimental scenario. In Situation 1 (S1), the outer and intermediate channels are closed at both ends, with the central channel serving as the sole flow path (left inlet, right outlet). In Situation 2 (S2), the inlets are located on the left side of the outer channels, while the central and intermediate channels remain closed on the left; flow exits through the right side of all channels. Finally, in Situation 3 (S3), only the left side of the intermediate channels is closed, with inlets positioned at the left of both the central and outer channels and all right-side ports acting as outlets. These configurations were established based on the potential operational modes of the proposed microfluidic device.

As illustrated in Figure 1E–G, different inlet and outlet flow configurations were considered, in which the volumetric flow rates applied at the inlets were kept constant within each condition at levels of 0.5, 1.0, and 5.0 μL/min (8.3, 16.7, and 83.3 × 10^−12^ m^3^/s, respectively), with an estimated Reynolds number of 1.75 at the inlet under the highest simulated flow, calculated using the hydraulic diameter (DH = 100 μm) of the 100 × 100 μm square inlet channel, a maximum fluid velocity of 0.0176 m/s, a fluid density of 998.2 kg/m^3^, and a dynamic viscosity of 0.001 Pa·s [22]. This condition corresponds to the highest Reynolds number considered in the present study, as it is associated with the maximum imposed inlet flow rate. This value therefore characterizes the maximum inlet-flow condition and should not be interpreted as the local Reynolds number throughout the different regions of the device. In all cases, the outlets were modeled with a zero-pressure condition, assuming fully open outlets. The working fluid used in the simulations was water, taken from the COMSOL Multiphysics® version 6.4 material library, assumed to be a continuous, incompressible, Newtonian, and isothermal fluid with a density of 998.2 kg/m^3^, a dynamic viscosity of 0.001 Pa⋅s, and a constant system temperature of 293.15 K. Water was selected as a simplified reference fluid to standardize the hydrodynamic analysis while avoiding composition-dependent variations associated with different culture media formulations. Gravity was neglected, and no fluid–structure interaction was considered. The channel walls and cell-sized probe surfaces were modeled as rigid and impermeable and assigned a no-slip boundary condition. The interconnecting micropores were explicitly represented in the device geometry and were not treated as a homogenized porous medium. No rotating reference frame or turbulence model was employed. The reference pressure was set to 1 atm.

### 2.2. Simulated Conditions for the Probe Cells

Additionally, as detailed in Figure 1B, three rigid and inelastic hemispherical structures with a radius of 10 μm (Cells 1, 2, and 3) were inserted as cell-sized geometrical probes, as computational surrogates, with the aim of evaluating the local distribution of WSS under different flow profiles. These rigid probes were used exclusively as geometric surrogates for mapping the local hydrodynamic field and were not intended to reproduce the deformability, adhesion behavior, or mechanical response of living cells. Each probe was modeled as a wall-attached hemisphere positioned on the bottom surface of the device, rather than as a complete sphere intersecting the wall. The hemisphere base was located on the bottom plane (z = 0), with the apex positioned 10 μm above the geometric center along the z-axis. The geometric centers of these hemispheres were positioned at coordinates (5, 0.7, 0) for Cell 1, (5, 1.8, 0) for Cell 2, and (5, 2.7, 0) for Cell 3. For each probe structure, an analysis point was defined at the apex of the surface (z = 0.01 mm), identified as Cell 1, Cell 2, and Cell 3.

**Figure 2:**
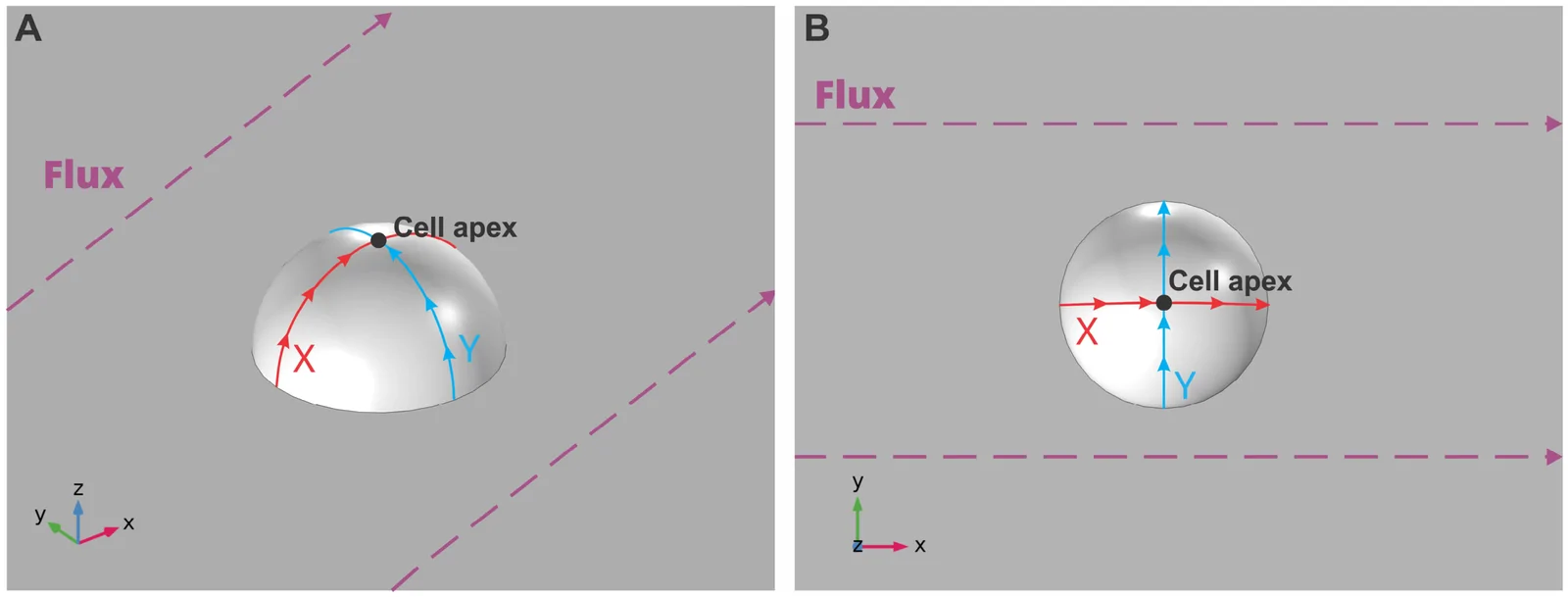
Determined cut lines in the surface of the cell-sized hemispherical probe structure, with the flow direction indicated by purple dotted lines. The horizontal cut line (red) is defined along the x-coordinates (flow direction), and the vertical cut line (blue) is defined along the y-coordinates (transverse to the flow direction) with each respective direction of increase. (**A**) illustrates the isometric view of the probe structure, while (**B**) illustrates the top view of the probe structure.

In addition, cut lines tangential to the surface of each probe structure were defined in order to obtain the numerical profile of WSS along their extent, as illustrated in Figure 2A,B. For each probe, the semicircular arc (referred to here as the major arc) was defined along the probe contour from one endpoint to the opposite endpoint, passing through the probe apex. The arc coordinate increases from one endpoint to the other, with the apex located at the midpoint of the arc.

### 2.3. Mesh Parameters

After the model was designed, the geometry of the microfluidic device was imported into COMSOL Multiphysics^®^ version 6.3, which enables multiphysics simulations based on the finite element method. The three-dimensional geometry was discretized using unstructured meshes composed of tetrahedral elements, and a mesh independence study was conducted using the grid convergence index (GCI) method by analyzing the percentage variation in WSS between meshes (normal, finer and extremely refined), with a minimum element ratio of 1.3. The WSS results showed mesh convergence, with relatively small differences between the finer and extremely fine meshes and GCI values below 1% for all investigated cell apices. The mesh-independence, GCI, and mesh-quality analyses for the three flow configurations are reported in Supplementary Tables S1–S3. The mesh used for data extraction contained, on average, 3.8231(6) × 10^7^ elements, with maximum and mean skewness values of 1.00 and 0.69, respectively. According to the mesh-quality definition implemented in COMSOL Multiphysics^®^, values range from 0 for degenerated elements to 1 for perfectly regular elements; therefore, higher values indicate better element quality [39]. Additional mesh-quality statistics are also provided in Supplementary Tables S1–S3. Mesh quality was evaluated together with the GCI-based mesh-independence analysis, considering WSS at the probe apices and in the micropore regions, as detailed in the Supplementary Material.

### 2.4. Physical and Numerical Modeling

The characterization of hydrodynamic parameters within the microfluidic device was performed using the laminar flow module available in COMSOL Multiphysics^®^. All simulations were performed under steady-state conditions, considering the previously defined boundary conditions. Equation (1) presents the steady-state incompressible Navier–Stokes momentum equation implemented in the model.

(1) $$\rho \left(u\cdot \nabla \right)u=\nabla \cdot \sigma +F$$

with $\sigma =\left[-pI+\mu \left(\nabla u+{\left(\nabla u\right)}^{T}\right)\right]$. Where u represents the fluid velocity vector, ρ the density, p the pressure, μ the dynamic viscosity, *I* the identity tensor σ the Cauchy stress tensor, −pI the isotropic pressure contribution to the stress tensor, $\mu \left(\nabla u+{\left(\nabla u\right)}^{T}\right)$ the viscous stress tensor, and F the body force per unit volume, which was set to zero because body forces were neglected in the simulations.

The Navier–Stokes equations were solved using a P1 + P1 discretization scheme based on continuous piecewise linear functions for both velocity and pressure, incorporating streamline and crosswind diffusion along with non-isotropic diffusion for stabilization.

As an independent analytical benchmark of the numerical solution, the inlet velocity for S1 was compared with the theoretical solution for fully developed laminar flow in a square duct [22]. Considering the 100 × 100 µm inlet cross-section, the mean velocity was calculated as $u_{\text{mean}}=Q/A$, where Q is the volumetric flow rate and A is the inlet cross-sectional area. For fully developed laminar flow in a square duct, the theoretical maximum velocity was estimated as $u_{\text{max}}\;\approx 2.096u_{\text{mean}}$ [22]. The comparison was performed at the highest investigated inlet flow rate (5 µL/min).

To estimate the WSS values on the channel walls, Equation (2) was used [40].

(2) $$WSS=\mu \cdot \frac{d\gamma }{dt}$$

where μ corresponds to the dynamic viscosity of the fluid, and $\frac{d\gamma }{dt}$ represents the wall shear rate, corresponding to the tangential velocity gradient at the solid–fluid interface. WSS therefore represents the magnitude of the tangential viscous traction acting on the wall or probe surface, and is obtained by the built-in parameter manipulation: spf.sr⋅spf.mu.

Once the WSS values were determined for the entire geometry, the surfaces of the cell-sized hemispherical probe structures and their respective arcs (illustrated in Figure 2) were isolated within COMSOL Multiphysics^®^ for detailed analysis.

Using the data extracted from the apex of each probe structure (Figure 1B), the dependence of WSS on inlet flow rate was assessed using the linear relationship (WSS =a+b⋅FLUX), where *FLUX* is the inlet flow rate, is the y-intercept corresponding to the extrapolated WSS at zero flow, and is the slope, representing the change in apical WSS per unit increase in inlet flow rate.

The study was conducted using a stationary solver, with a relative tolerance of 0.001 (default by COMSOL), using the Algebraic Multigrid (AMG) solver with generalized minimal residual (GMRES) method with residual tolerance of 0.01, for the maximum number of iterations established at 1000, with solutions initialized from zero velocity. Each simulation was solved independently for the corresponding flow configuration and inlet flow rate, and convergence was achieved according to the specified solver tolerances.

Within this computational framework, the cell-sized hemispherical probe structures were used as standardized geometrical elements to quantify the local mechanical conditions generated under the different flow configurations. Their inclusion enabled the assessment of how device geometry, flow distribution, and operational conditions influence the WSS acting on cell-sized surfaces at specific regions of the microfluidic device. These rigid and fixed probe structures were not intended to reproduce the complete biological behavior of migrating cells or their interactions with neighboring cells and the extracellular matrix, but rather to provide a controlled and reproducible representation for the local quantification of flow-induced mechanical stimuli.

## 3. Results

The main parameters analyzed in this study were the distribution of flow velocity, pressure, and WSS throughout the entire microfluidic device, while WSS along the surfaces and major arcs of the cell-sized probe structures was evaluated to characterize the local mechanofluidic environment rather than to predict cellular behavior.

### 3.1. Computational Simulations

#### 3.1.1. Flow Velocity, Pressure and Wall Shear Stress Magnitudes

**Figure 3:**
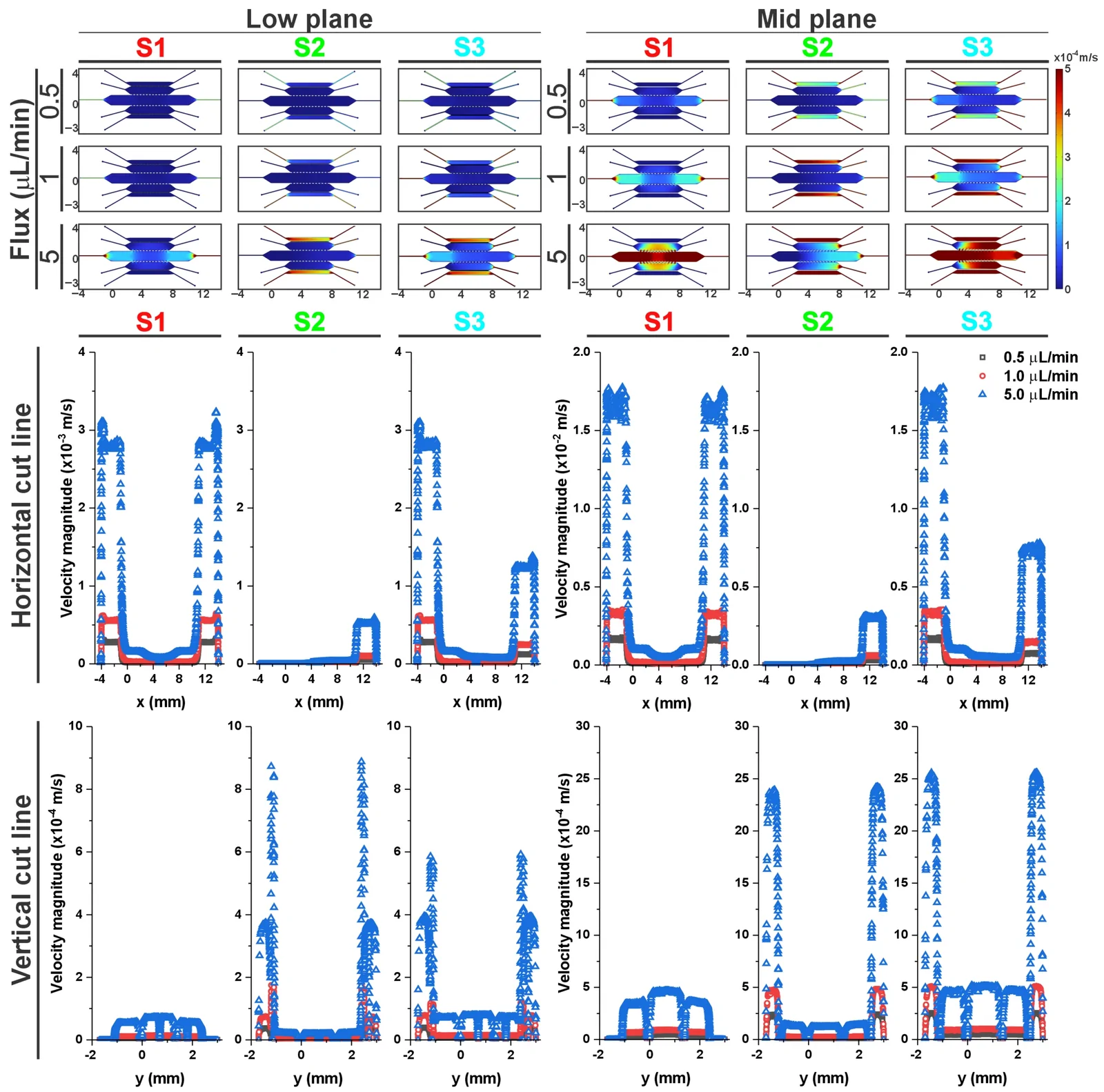
Velocity magnitude fields and numerical velocity profiles for Situations 1–3 (S1–S3). S1 corresponds to central-channel perfusion, S2 to lateral-channel perfusion, and S3 to combined central and lateral-channel perfusion. The top panels show the velocity magnitude fields for inlet flow rates of 0.5, 1.0, and 5.0 µL/min at the low (z = 0.004 mm) and mid (z = 0.05 mm) planes, using the same color scale for direct comparison. The middle panels show the velocity profiles along the horizontal cut line (y = 0.7 mm), and the bottom panels show the profiles along the vertical cut line (x = 5 mm). The numerical profiles are presented for both planes and all three inlet flow rates: 0.5 µL/min (black squares), 1.0 µL/min (red circles), and 5.0 µL/min (blue triangles).

Considering the three inlet flow rates (0.5, 1, and 5 μL/min) and the three manipulation scenarios idealized and described in Figure 1E–G, the fluid velocity distributions were obtained for the low and mid planes, as shown in Figure 3. In the same figure, numerical velocity values along the horizontal cut lines (middle portion of the figure) and the vertical cut lines of the device (lower portion of the figure), traced on both planes of analysis, are also presented.

The analysis of the velocity distributions demonstrates agreement with Poiseuille’s law: in the mid-plane, located at half the device height, higher velocities were observed compared to the low plane near the base. Under all the conditions evaluated, the lateral channels exhibited higher velocities than the central channel, apart from S1, in which there was no flow in the outer channels. Overall, velocity values ranged from 10^−4^ to 10^−2^ m/s throughout the device for all situations.

For S1 at 5 µL/min, the analytical benchmark yielded a mean inlet velocity of 8.33 × 10^−3^ m/s and a theoretical maximum velocity of approximately 1.75 × 10^−2^ m/s. The corresponding maximum velocity obtained from the CFD model was 1.76 × 10^−2^ m/s, resulting in a difference of approximately 0.8%. This close agreement supports the consistency of the numerical solution with the analytical estimate for the simplified inlet flow condition.

The 0.5 μL/min flow condition, represented by gray squares in the graphs of Figure 3, exhibited velocity values between 5 × 10^−5^ m/s and 1.7 × 10^−3^ m/s. The lowest values were consistently recorded at the center of the central chambers in all simulations, while the highest values, reaching 1.7 × 10^−2^ m/s, were observed at the device inlet and outlet for S1. The profile shown in the top graphs of Figure 3 indicates that the flow intensities in the low plane of the device are very small for the adopted scale. On the other hand, in the mid-plane, the intensities are compatible with the scale, correctly representing the regions of higher flow. In S1, the flow intensity is almost entirely concentrated at the inlet and outlet of the central channel, with the lateral channels exhibiting no significant flow. In S2, the relevant flow is concentrated in the lateral channels, with the central channel displaying relatively low intensity. S3 is the only one in which both the lateral and central channels indicate high flow intensity, corroborating the initial conditions proposed by the simulation.

S3 exhibited behavior similar to S1 for x-coordinates below 4 mm. However, for x-values exceeding this threshold, a flow reduction of approximately one half was observed. This decrease is attributed to pressure redistribution resulting from the opening of the intermediate channels.

In S2, the central channel was only influenced by the flow for x > 4 mm. Since there was no direct flow into this specific channel, its outlet flow rate was lower than in the other configurations, particularly when compared to the flow observed in the lateral channels.

Regarding the vertical cut lines, the velocity distribution within the central channel was nearly identical between S1 and S3 for the y-interval ranging from approximately −1 mm to 3 mm. In S1, only the central outlet was open, while the remaining outlets were closed; therefore, no independent flow was imposed through the intermediate channels. However, because these channels are physically connected to the central region, local fluid redistribution occurred before the flow converged toward the central outlet, resulting in small non-zero local velocities. In S3, the contribution of flow from the lateral channels toward the central region was strongly attenuated by the porous barriers, resulting in a similar velocity profile within this interval.

In contrast, S2 displayed a distinct behavior, as it was the only condition characterized by the absence of direct flow entry into the central channel.

In the lateral channels (y < −1 mm and y > 3 mm), the distribution patterns remained consistent across all studied situations. The highest velocities in both planes were recorded in S1, specifically at the inlet regions along the horizontal line.

Regarding the horizontal cut line graphs, the central regions of the device exhibit the lowest velocity values, while the highest values are concentrated in the fluid inlet and outlet regions. For the vertical cut lines, it is observed that the flow intensity in the central channel does not differ substantially from that in the intermediate channels. However, in S2 and S3, the flow intensities in the lateral channels are higher than those in the central channels due to the flow restriction imposed by the presence of micropores and the smaller cross-sectional area of the lateral channels compared with the central channel. These geometrical features reduce the effective cross-sectional area available for flow and contribute to the higher velocities observed in the lateral channels.

**Figure 4:**
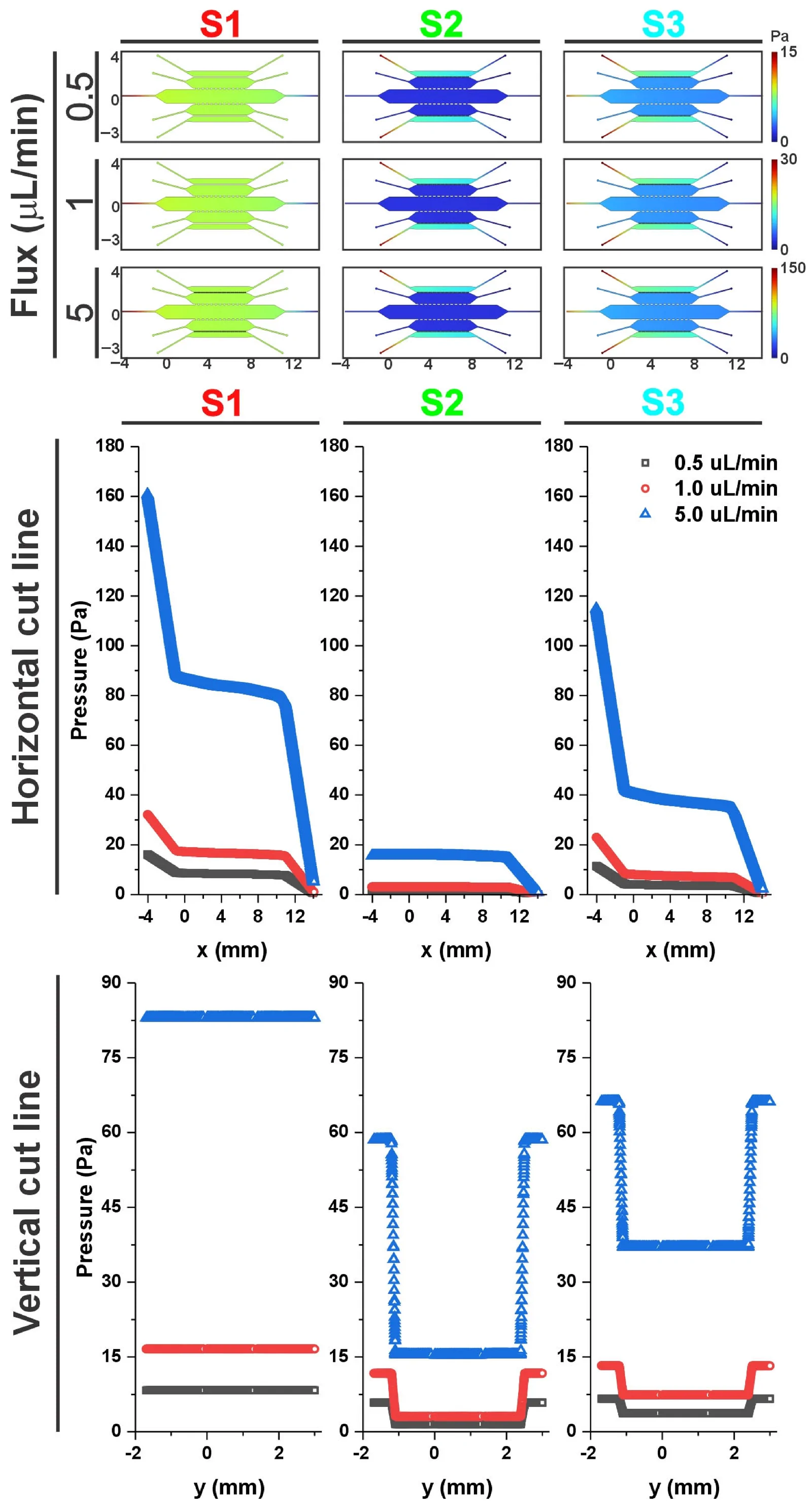
Pressure fields and numerical pressure profiles for Situations 1–3 (S1–S3). S1 corresponds to central-channel perfusion, S2 to lateral-channel perfusion, and S3 to combined central and lateral-channel perfusion. The top panels show the pressure distribution in the low plane (z = 0.004 mm) for inlet flow rates of 0.5, 1.0, and 5.0 µL/min. Different color scales (0–15, 0–30, and 0–150 Pa) were used for the three inlet flow rates to accommodate the corresponding pressure ranges and improve visualization. Pressure values are reported as gauge pressure relative to the zero-pressure outlet boundary condition. The middle panels show the pressure profiles along the horizontal cut line (y = 0.7 mm), and the bottom panels show the profiles along the vertical cut line (x = 5 mm). The numerical profiles are presented for 0.5 µL/min (black squares), 1.0 µL/min (red circles), and 5.0 µL/min (blue triangles). Results are shown for the low plane only, as the pressure distributions were identical to those obtained in the mid-plane.

The pressure analysis was conducted in a manner analogous to the study of velocity distributions, considering the low and mid-planes, as well as the values obtained along the horizontal and vertical cut lines, shown in the middle and lower portions of Figure 4, respectively. Unlike the velocity distribution, the spatial pressure distribution maintained the same pattern across the different flow conditions, being directly proportional to the increase in the applied flow rate, as illustrated in Figure 4. This distribution is practically independent of the height of the plane analyzed; therefore only the low plane was presented as representative for the pressure analysis.

Along the horizontal cut lines, a decreasing pressure gradient in the flow direction is observed, which is consistent with the boundary conditions imposed in the simulation, where pressure is zero at the outlets and maximum at the inlets. Along the vertical cut lines, higher pressures can be identified in the outer channels across all analyzed conditions. This behavior is attributed to the smaller cross-sectional diameter of the lateral channels, which results in greater flow resistance and, consequently, higher pressure accumulation.

The pressure analysis revealed a dependence on the inlet and outlet configuration of the device. In S1, pressure was nearly constant throughout the device, except in the inlet and outlet regions, which correspond to the maximum and minimum values, respectively. Due to the closure of all lateral outlets in this configuration, pressure becomes uniform across the internal region, indicating flow restriction. In S2, the highest-pressure values are observed at the inlets of the lateral channels, corresponding to flow injection into the device. The remainder of the device exhibits lower mean pressure values than those observed in S1, since the greater number of open outlet channels in S2 facilitates pressure relief. In S3, the behavior is similar to that of S2, but with higher-pressure values, resulting from the addition of an extra flow inlet channel in the device.

The pressure reduction behavior induced by the increased opening of channels is corroborated by the horizontal cut line graphs. For the 5 μL/min flow condition, the inlet pressure values are ~160 Pa in S1, decreasing to approximately 120 Pa in S3. When comparing the central region of the device among the three situations, S1 exhibits the highest-pressure values (~85 Pa), followed by S3 (~40 Pa) and, lastly, S2 (~15 Pa). This difference is attributed to the number of open outlet channels and to the initial no-flow conditions imposed in S2.

In S2, pressure values along the horizontal cut line reached a maximum of ~20 Pa, even under the highest flow rates. This is due to the line’s positioning outside the fluid inlet region. Conversely, in S3, the peak observed value reached ~120 Pa, coinciding with the flow entry into the central channel. This configuration also exhibited a higher contribution of regions with zero pressure (5 outlets), which accounts for the discrepancy compared to S1. The latter reached a higher maximum pressure of ~160 Pa, characterized by a more intense pressure gradient resulting from the presence of only a single outlet.

**Figure 5:**
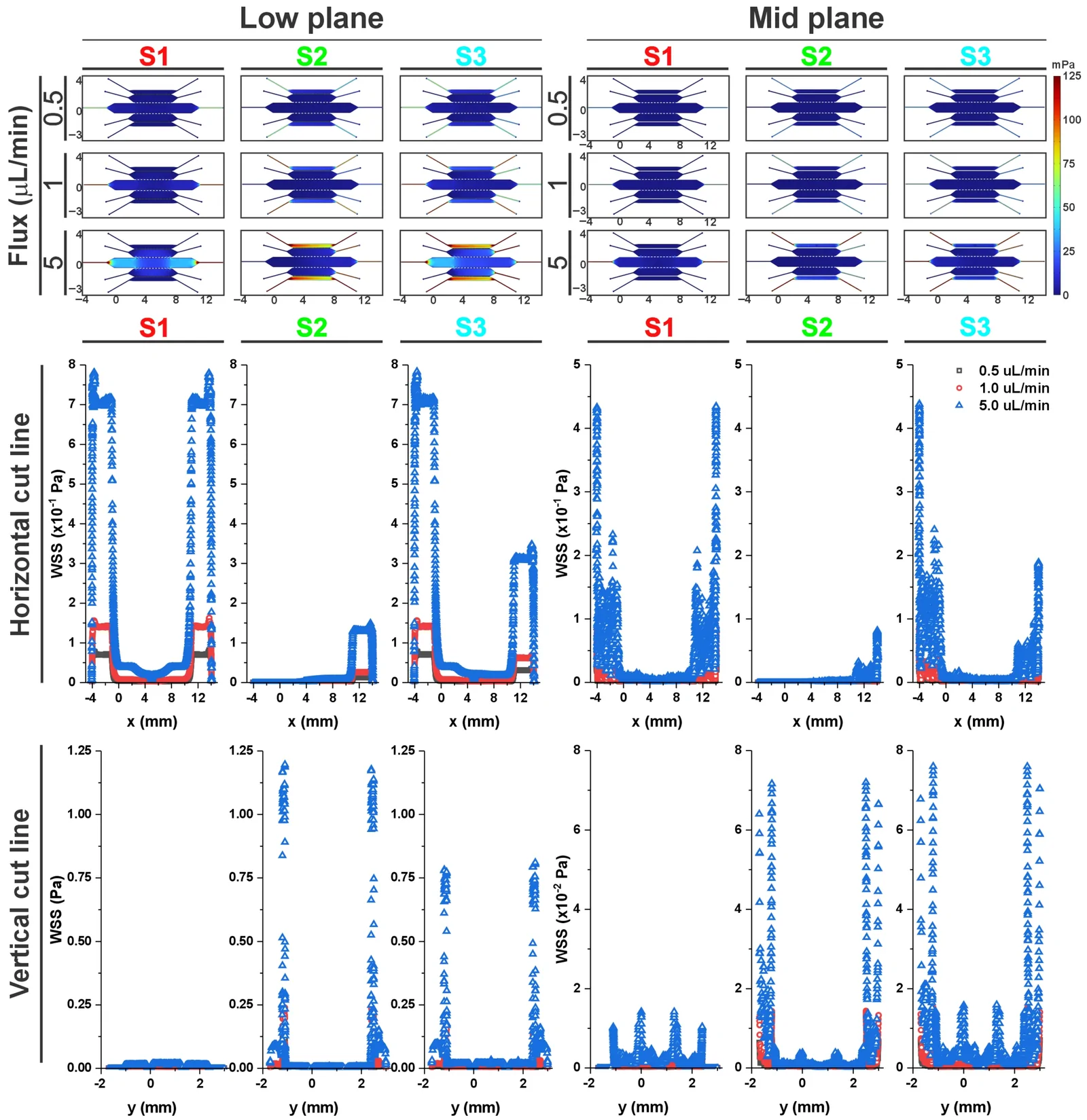
Wall shear stress (WSS) distributions and numerical WSS profiles for Situations 1–3 (S1–S3). S1 corresponds to central-channel perfusion, S2 to lateral-channel perfusion, and S3 to combined central and lateral-channel perfusion. The upper panels show the WSS distributions for inlet flow rates of 0.5, 1.0, and 5.0 µL/min in the low plane (z = 0.004 mm) and mid plane (z = 0.05 mm), using a common color scale from 0 to 125 mPa for direct comparison. The middle panels show WSS profiles along the horizontal cut line (y = 0.7 mm), and the lower panels show profiles along the vertical cut line (x = 5 mm). The numerical profiles are presented for 0.5 µL/min (black squares), 1.0 µL/min (red circles), and 5.0 µL/min (blue triangles). WSS values are expressed in mPa in the color maps and in Pa in numerical profiles, with the corresponding scale factors indicated on the axes.

In order to investigate the impact of different flow conditions on the local mechanical microenvironment, WSS profiles were analyzed on the two previously defined planes, as illustrated in Figure 5. In the same figure, the numerical values obtained along the horizontal cut lines (middle portion of Figure 5) and the vertical cut lines (lower portion of Figure 5) are also presented, allowing a quantitative and spatial evaluation of WSS. WSS is higher in regions close to the channel walls, particularly at the base of the chip, compared to the mid plane. There is a direct relationship between flow rate and WSS values: regions with higher flow exhibit increased WSS, concentrated along the walls adjacent to the fluid pathway. The color scale in Figure 5 was limited to enhance the visualization of spatial WSS differences; therefore, the maximum values for S2 and S3 at 5 µL/min exceed the displayed range. A version with an expanded WSS scale for the low plane is provided in the Supplementary Material (Figure S1).

The WSS within the device exhibited a behavior opposite to that observed for velocity, with higher values recorded in the low plane than in the mid plane. According to the color scale in Figure 5, only a few regions reached the highest values. Elevated WSS values are evident in the lateral channels of the device for the 5 μL/min flow condition in the low plane. For the other flow rates and in the mid plane, WSS values did not reach the highest scale adopted.

The WSS values of the middle plane showed greater variability, while the lower plane showed smoother profiles. S2 showed higher WSS in the region near the central outlet, attributed to the increased flow intensity in that area. S3, on the other hand, exhibited an almost symmetrical distribution, similar to S1, differing mainly in the increased number of exits, which resulted in a higher total flow. Analysis of the horizontal cut lines reveals that fluid WSS is higher at the inlet and outlet regions of the device. For the 5 μL/min flow rate, values in these regions range from approximately 0.4 Pa to 0.8 Pa for S1 and S3 (in both the mid and low planes), respectively, whereas S2 exhibits lower values, approximately 0.2 Pa (Figure 5).

From the vertical cut line results, it is observed that in the central region of the device, the highest WSS values obtained for S1 did not exceed 0.1 Pa. However, in the micropore regions, WSS reaches substantially higher values: the micropores in S2 attained the maximum value recorded in the simulations, 1.25 Pa, while the micropores in S3 reached approximately half of this value, around 0.75 Pa. These elevated WSS values are consistent with the local flow acceleration associated with the reduced cross-sectional area of the micropores. Mesh-refinement analysis performed for S2 showed convergence of the WSS distribution in the micropore region across the evaluated meshes (Supplementary Figure S2). Outside the micropores, the lateral channels in S2 and S3 present values close to 0.125 Pa.

The study showed that for the computer simulations, the values of velocity, pressure, and WSS presented a direct proportional relationship as a function of the applied flow rates, regardless of the flow configuration adopted, as expected when numerically solving the Navier–Stokes equations, which enables the estimation and evaluation of the device’s mechanofluidic behavior under the different conditions assessed in the present study. It was observed that the maximum values of these parameters consistently followed the ratio 1:2:10, corresponding to the proportions of the inlet flow rates (0.5, 1 and 5 μL/min).

Although this proportionality is analytically expected for laminar flow, the main contribution of the present analysis lies in the systematic comparison of different inlet/outlet configurations within the same three-dimensional microdevice. Under the same imposed flow rates, S1, S2, and S3 generated distinct spatial distributions and local magnitudes of velocity, pressure, and WSS, demonstrating the influence of operational configuration on the local mechanical microenvironment.

#### 3.1.2. Wall Shear Stress Magnitudes on the Cell-Sized Probe Structures

**Figure 6:**
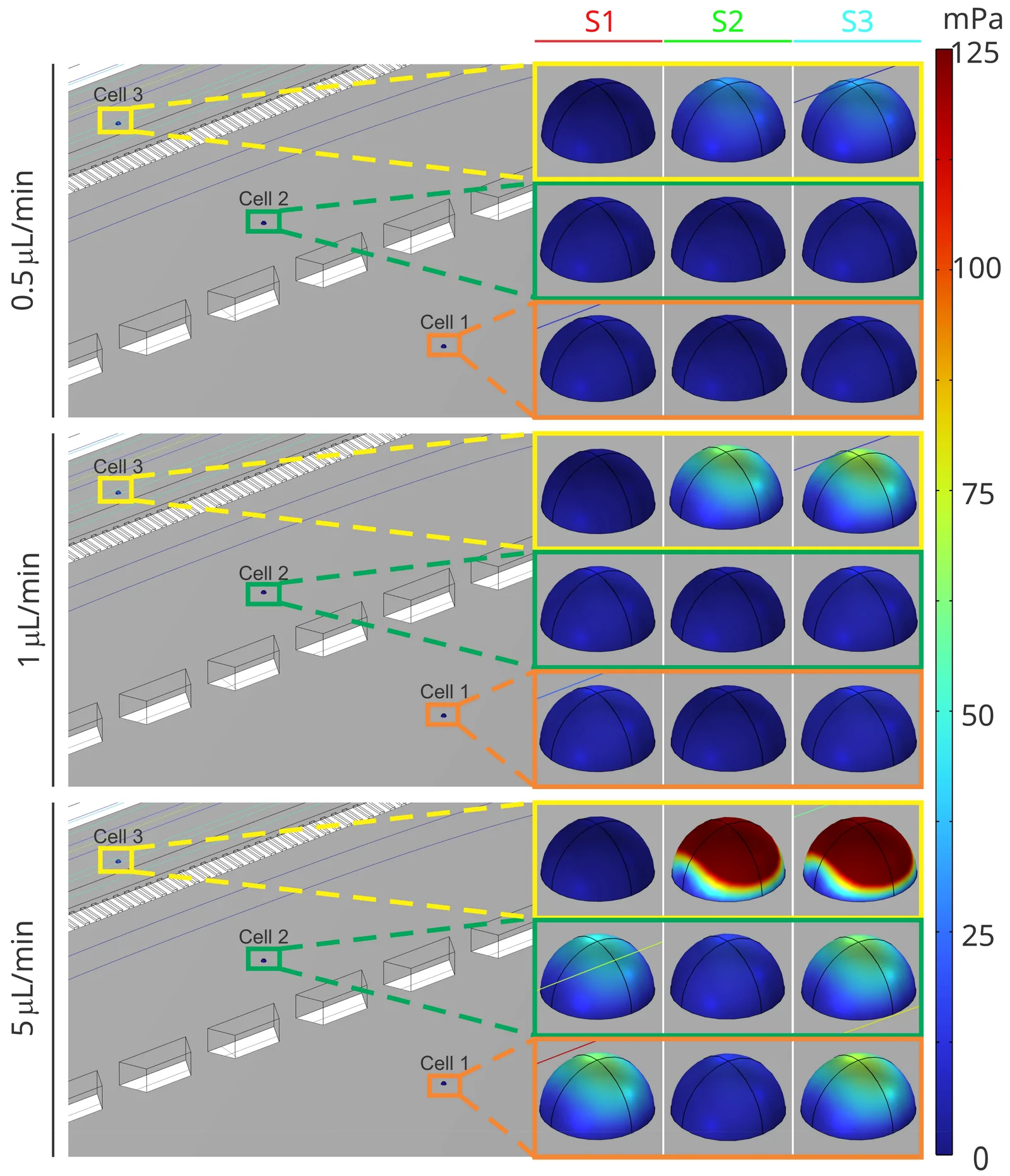
Spatial distribution of WSS on cell-sized probe surfaces across multiple experimental parameters. This figure provides a comprehensive computational analysis of the WSS magnitude, measured in millipascals (mPa), acting upon the surfaces of three representative cell-sized probe structures (Cell 1, Cell 2, and Cell 3) within a microfluidic environment. The data are organized vertically by increasing flow rates (0.5 µL/min, 1 µL/min, and 5 µL/min) illustrating a proportional escalation in mechanical loading as fluid velocity rises. On the left, 3D isometric views of the microchannel identify the specific spatial coordinates of each cell cluster, the lateral channel (yellow), the internal channel (green), and the central channel (orange). The right-hand matrices detail the stress profiles for each situation (S1, S2, and S3), revealing how local flow dynamics and cell positioning influence the uniformity of WSS distribution. A standardized colorimetric scale ranging from 0 mPa (dark blue) to 125 mPa (dark red) is applied across all panels to facilitate direct comparison.

Considering the relevance of WSS as a mechanical stimulus in cell-based microfluidic systems, its distribution was analyzed in three specific regions of the chip, simulating the presence of cell-sized hemispherical probe structures with a diameter of 20 µm. The probe structures were modeled as rigid and inelastic structures for the purpose of quantifying local WSS values. The results for all flow and manipulation conditions are presented in Figure 6.

With the color scale limited to 125 mPa, it is observed that in S1, the 0.5 and 1 μL/min flow rates do not present values close to 125 mPa on any of the cells, whereas for the 5 μL/min flow rate, Cells 1 and 2 show indications of WSS variation over their surfaces. In S2, a flow rate of 1 μL/min is already sufficient to indicate WSS variation on Cell 3; however, at 5 μL/min, Cell 3 experiences WSS values exceeding the 125 mPa scale, while Cells 1 and 2 appear to exhibit values well below 125 mPa. In S3, the 0.5 and 1 μL/min flow rates are very similar to those observed in S2, differing at the 5 μL/min condition, in which Cells 1 and 2 exhibit behavior comparable to that observed in S1.

**Figure 7:**
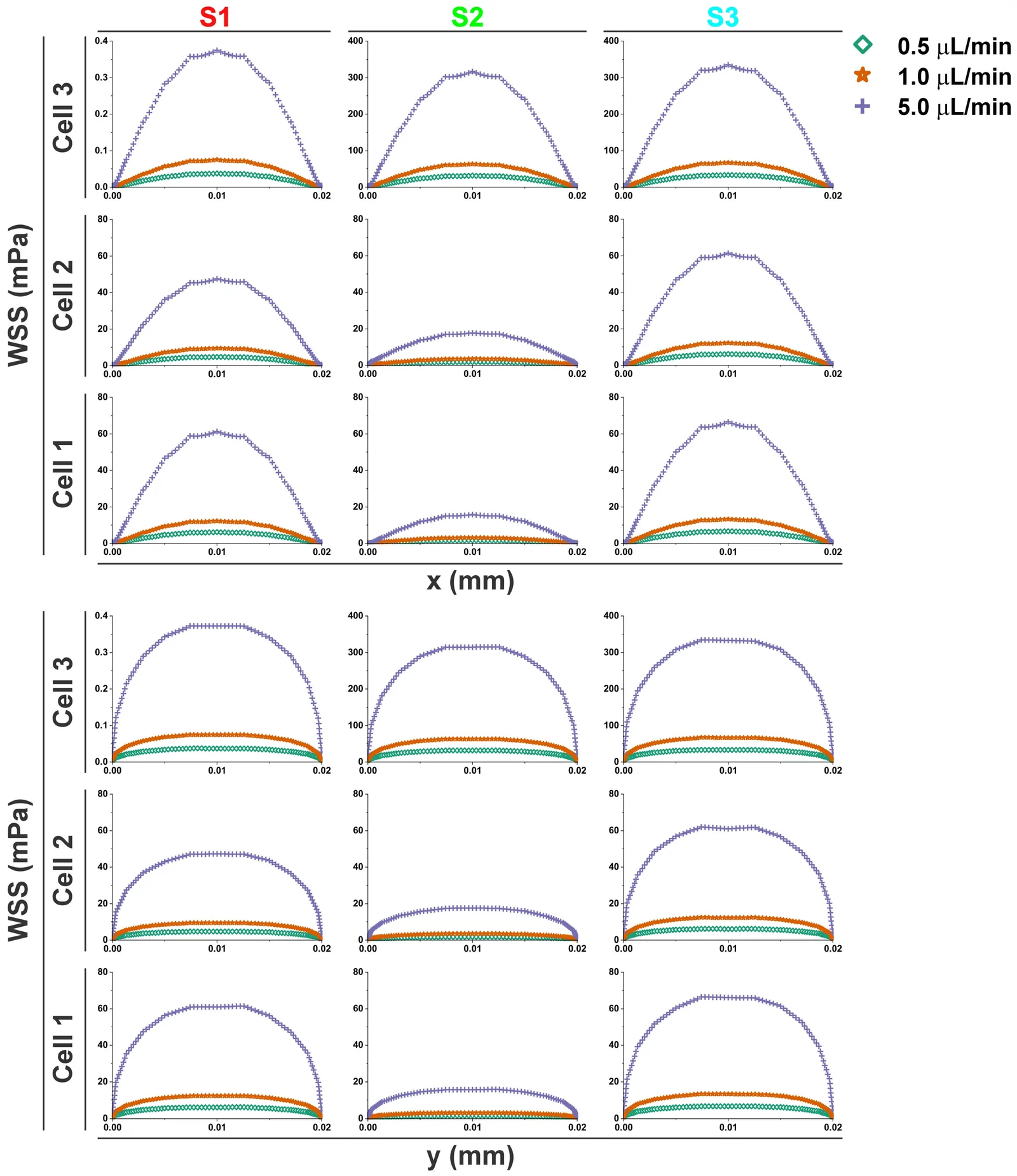
WSS along the semicircular probe arcs of the cell-sized probe structures in the x-axis and y-axis orientations for each situation (S1, S2, and S3). The top panel displays WSS values along the horizontal (x) cut line, while the bottom panel shows the vertical (y) cut line, as defined in Figure 2. Results for different inlet flow rates are represented by 0.5 μL/min in green diamonds, 1 μL/min in orange stars, and 5 μL/min in purple plus signs. The coordinate increases from 0 mm at one endpoint of the semicircular probe arc to 0.02 mm at the opposite endpoint, with 0.01 mm corresponding to the probe apex.

Complementary to the analysis of surface WSS distribution, numerical values were obtained along the semicircular probe arcs of the cell-sized probe surface, considering the two orientations shown in Figure 2. This approach enabled the assessment of local variations that are not captured by the color scale in Figure 6. The corresponding numerical results are presented in Figure 7, in which the coordinate along the semicircular probe arc was expressed using a diameter-based coordinate ranging from 0 to 0.02 mm, with 0.01 mm corresponding to the probe apex, allowing comparisons among different regions of the surface. In addition, we have provided a statistical summary table including the mean, minimum, median, maximum, and 95th percentile of the WSS values on the probe surface, as presented in Supplementary Material S4–S6.

The WSS distribution observed around the cell-sized probe structures exhibits symmetry in both the vertical and horizontal arcs, with the maximum value consistently located at the apex of the probe structure, reflecting the local fluid-flow distribution around the cell contour. This symmetry results in similar local velocity gradients on opposing sides of the probes, producing comparable WSS values at corresponding positions along the cell contours, with the maximum WSS consistently occurring at the apex of each probe structure. This behavior reflects the local flow field around the fixed hemispherical probes and is not a consequence of plotting both sides together. Variations in flow rate demonstrate a direct proportional relationship with WSS values. Increases in flow rate follow a 1:2:10 ratio (corresponding to 0.5, 1, and 5 μL/min), and the same proportion is reflected in the WSS values. For example, in Cell 2 under S1, a flow rate of 0.5 μL/min results in approximately 5 mPa at the cell apex; increasing the flow rate to 1 μL/min raises this value to approximately 10 mPa; and a flow rate of 5 μL/min reaches 50 mPa. Considering the 5 μL/min flow condition, in S1, Cells 1 and 2 exhibit similar WSS values, ranging between 50 and 60 mPa. In contrast, Cell 3 records extremely low values, around 0.4 mPa, due to the absence of effective flow in that specific region under this condition. In S2, the WSS at the apex of Cell 3 reaches approximately 300 mPa. Conversely, Cells 1 and 2 exhibit reduced WSS, approximately 20 mPa, reflecting a decrease in the mechanical stimulus delivered to the central cells because of flow rearrangement. S3, in turn, presents a WSS profile that is essentially a combination of S1 and S2: Cell 3 reaches approximately 300 mPa, while Cells 1 and 2 maintain more moderate values, around 50 mPa.

The highest WSS values were recorded under the condition of a flow rate of 5 μL/min, indicating an approximately linear relationship between flow rate and WSS. Cells 1 and 2 showed values between 20 and 60 mPa for S1 and S2, while Cell 3 reached values between 300 and 400 mPa for S2 and S3, respectively, reinforcing that Cells 1 and 2, in general, were exposed to lower WSS values in all situations.

**Figure 8:**
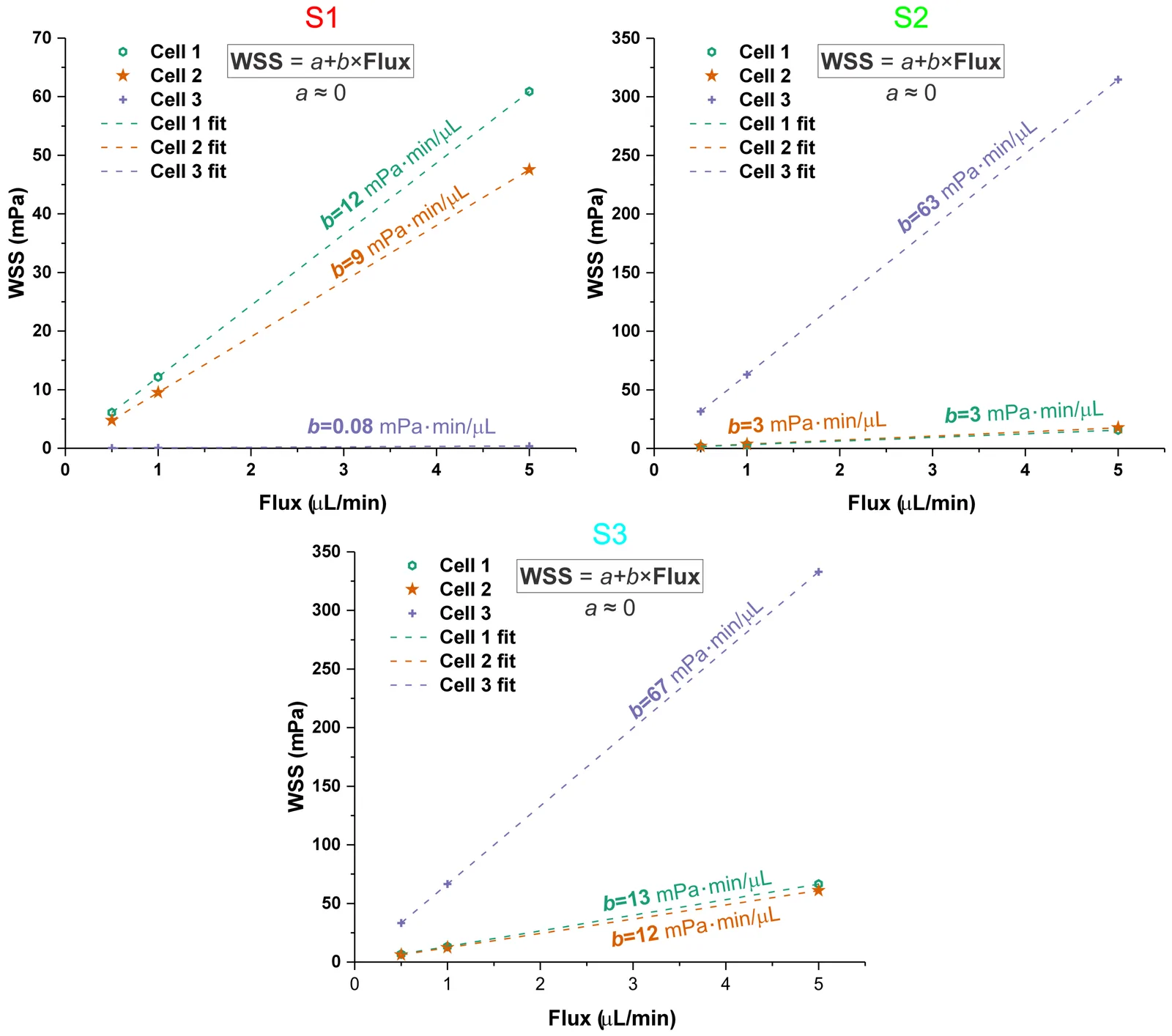
Linear relationships between WSS and the applied flow rate for Situations 1–3 (S1–S3). Green diamonds, orange stars, and purple plus signs represent Cells 1, 2, and 3, respectively. Dashed lines of the corresponding colors indicate the linear fits for each cell.

Based on the WSS values obtained at the apex of each cell-sized probe structure, a proportional relationship between the applied flow rate and WSS was observed for the evaluated conditions. This relationship was influenced by the inlet flow conditions and the spatial position of each probe structure. To characterize this proportionality, the data were represented by linear relationships of the form WSS=a+b×Flux (where *a* represents the intercept and *b* the slope) and were derived to describe the WSS behavior for different flow rates (*Flux*) for proportionality observations, as shown in Figure 8. These relationships were used as proportionality checks rather than for statistical validation or assessment of predictive accuracy. The intercepts were numerically negligible, consistent with the expected near-zero WSS at zero imposed flow and with the proportionality between WSS and flow rate under the simulated conditions. Detailed parameters and numerical errors are reported in Supplementary Tables S7–S9.

In all adjustments performed, the coefficient of determination (R^2^) was equal to 1. This result is expected, as the data were obtained through computational simulations. Furthermore, in all equations, the intercept was between the order of 10^−10^ and 10^−7^ mPa, making it negligible compared to the slope.

The analysis of the slopes, which represent the progression rate of WSS in relation to the flow (mPa∙min/µL), revealed differences between the situations. In S1, the highest slope obtained was 12 mPa∙min/µL, corresponding to Cell 1, which is the direct flow inlet region. On the other hand, the value presented for Cell 3 (0.08 mPa∙min/µL) proved to be negligible in comparison.

This pattern is reversed in S2, where Cell 3 presented the highest value for the slope, reaching 63 mPa∙min/µL, while the adjustments performed for Cells 1 and 2 resulted in slopes of 3 mPa∙min/µL. S3, in turn, presented results similar to S2, but with slightly higher values, with the maximum slope obtained for Cell 3 (67 mPa∙min/µL). The remaining slopes, corresponding to Cells 1 and 2, were 12 mPa∙min/µL. This result indicates a behavior similar to that of Cell 1 in S1, but with a notable increase in progression for Cells 2 and 3 compared to S1 and S2.

The progression of WSS was greater in regions directly associated with flow inlet or flow convergence. In regions without direct flow inlet, as observed for Cell 3 in S1, the increase in WSS as a function of flow rate was negligible. In configurations S2 and S3, the combination of flow inlets in different channels modified the WSS distribution, with greater influence in the regions corresponding to Cells 2 and 3. On the other hand, Cell 1 did not show significant variation between S1 and S3, indicating similar behavior in these two conditions.

#### 3.1.3. Literature-Based Validation of Simulated Parameters

The magnitude and behavior of the simulated parameters were compared with previous computational and experimental studies involving microfluidic and organ-on-a-chip platforms. The direct proportionality observed between inlet flow rate and WSS in all situations (S1–S3) is consistent with the laminar flow regime reported by Kim et al. [30] and Pisapia et al. [31], who also described increases in WSS as a function of flow rate and channel geometry. Similar trends have also been reported in a mechanobiological study employing a microfluidic system to characterize flow-induced mechanical stimuli associated with cell behavior and migration processes [38].

The WSS values obtained at the cell-sized probe structures, ranging from approximately 0.4 to 400 mPa depending on flow rate and configuration, fall within the same order of magnitude reported by Shemesh et al. [32] for adherent cells in microfluidic devices, as well as by other CFD-based microfluidic design studies [36,37]. These values fall within ranges of mechanical stimuli reported in mechanobiology and cell migration studies, in which fluid-induced stresses have been associated with changes in cytoskeletal organization, mechanotransduction, adhesion, and migration behavior [38,41]. However, these comparisons are intended only to contextualize the simulated WSS exposure and should not be interpreted as evidence that the rigid probe structures reproduce the mechanical response of living cells.

Furthermore, the higher WSS values concentrated near micropores and channel inlets/outlets reproduce spatial patterns commonly described in the microfluidic CFD literature [30,31,37]. These regions are expected to exhibit local flow acceleration and increased shear gradients due to geometric restrictions, which further supports the consistency of the simulated flow behavior with previously reported CFD studies involving microfluidic devices.

Taken together, the agreement between the present results and previously published studies, both in terms of parameter magnitude and spatial distribution patterns, supports the reliability of the numerical predictions and indicates that the predicted hydrodynamic behavior of the device is consistent with established computational and experimental observations reported in the literature.

## 4. Discussion

The computational approach used in this study characterized the mechanical microenvironment generated within the three-dimensional microfluidic device under different inlet/outlet configurations and flow rates. Rigid hemispherical probes were used as standardized cell-sized geometrical elements to quantify local WSS without reproducing the biological behavior of migrating cells. These data may support the design and optimization of future cell migration experiments [31,42,43,44,45,46].

This study demonstrated that device geometry and inlet/outlet conditions determine the spatial distribution of hydrodynamic forces, particularly WSS, creating mechanically heterogeneous microenvironments [17,47]. Such heterogeneity is relevant to cell migration and mechanobiological studies because different regions may experience distinct mechanical stimuli, even under the same global flow rate [33,48].

The dimensions adopted here are consistent with microfluidic platforms used for cell migration and transmigration studies, providing confined flow conditions while maintaining compatibility with optical imaging and PDMS–glass fabrication [30,31,36,43,49,50,51,52,53,54,55].

Although velocity and pressure distributions were consistent with laminar microscale flow [22,40], interconnected channels, micropores, and different inlet/outlet configurations generated local flow redistributions requiring three-dimensional characterization [30,56,57,58].

From an experimental perspective, the simulated flow rates investigated in this study could be achieved using recently developed passive perfusion strategies. Hydrostatic pressure-driven modular systems incorporating resistance channels enable tunable and sustained perfusion without external pumps, while low-cost pumpless assemblies based on hydraulic resistance can support long-term microfluidic culture. More recently, the CHARM system has demonstrated stable gravity-driven flow through automatic regulation of the hydraulic head, providing an additional approach for maintaining controlled perfusion over extended periods [59,60,61].

Interstitial transport through the extracellular matrix can be understood within the framework of flow through porous media [62], while the resulting mechanical forces may contribute to the regulation of tumor growth and therapeutic response [63].

Importantly, the WSS characterized in this study should not be interpreted as a direct representation of interstitial flow in vivo. In native tissues, cells embedded within three-dimensional extracellular matrices are predominantly exposed to pressure-driven interstitial transport, whereas perfused microfluidic channels generate shear forces at solid boundaries [52,53,54,55,56,57,58]. Therefore, WSS is considered here as an engineered and experimentally controllable mechanical stimulus for characterizing the device-generated microenvironment, rather than as a direct surrogate for physiological interstitial flow [59,60,61,62,63,64,65,66]. Accordingly, the present device generates an engineered perfusion environment with measurable WSS and does not reproduce the complete mechanical environment of native interstitial flow.

Regarding WSS distribution, the non-uniformity observed along the device can be attributed to asymmetry in flow distribution, resulting from different combinations of open and closed channels, and to geometric differences, as the internal channels present a larger cross-sectional area than the lateral channels [33,48]. Regions near the inlets, outlets, and interconnecting pores exhibit higher WSS values, whereas internal regions experience lower WSS. Such regions may serve as experimentally testable microenvironments, as spatial variations in mechanical stimuli have been associated with cell polarization and cytoskeletal reorganization [12,14], cell adhesion and integrin-mediated signaling [64]. and other mechanotransduction-related responses [65,66,67,68].

The analysis of the pore regions shows that local reductions in cross-sectional area intensify WSS, even under moderate flow conditions. Although this behavior is consistent with fundamental hydrodynamic principles, it differs from observations reported in other microfluidic devices, mainly due to differences in chip geometry and interconnecting pores [31]. In this context, the engineering of microfluidic devices can be used as a tool to generate controlled gradients of mechanical stimuli, providing a quantitative mechanical framework for the design and interpretation of subsequent experimental studies investigating cell behavior.

The interaction between fluid flow and the cell surface leads to the development of WSS that, depending on its magnitude and the specific cellular context, may influence processes such as cell adhesion, migration, and viability [15]. In this work, cell-sized hemispherical probe structures were represented as rigid, fixed, and inelastic geometries in order to isolate and quantify the magnitude of the WSS imposed by the flow, without the influence of active cellular responses. This approach reduces computational cost and enables a systematic analysis of the hydrodynamic conditions within the device. Similar simplified geometric representations have been previously used in computational studies to investigate local flow and hydrodynamic conditions [65,66,69]. In contrast, models that incorporate deformable cells are more suitable for investigating phenomena such as cell deformation and mass transport; however, they require greater computational complexity and cell-type-specific parameters [67,68,70,71].

**Figure 9:**
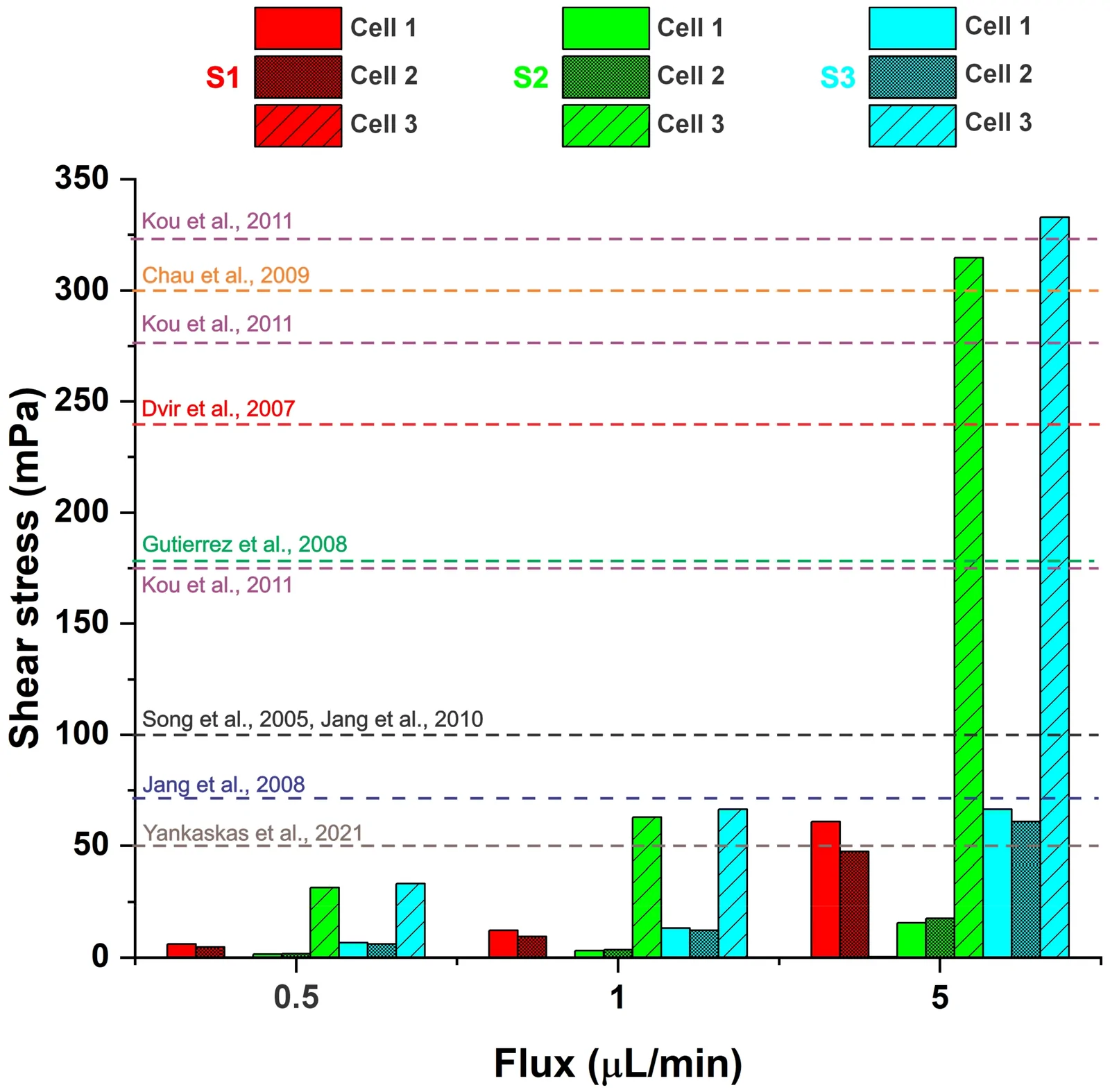
WSS at the cell apex under the different simulated scenarios. Results for S1, S2, and S3 are represented in red, green, and cyan, respectively. Data for each cell is distinguished by fill patterns: solid bars (Cell 1), dotted bars (Cell 2), and diagonal hatched bars (Cell 3). Dashed colored lines indicate literature-reported WSS values or ranges associated with specific cellular responses and are included as reference points for contextual comparison rather than as universal mechanobiological thresholds. The reported mechanobiological responses are changes in fibroblast migration [79], enhanced osteoblast activity [75], endothelial cell elongation [72], renal cell polarization and cytoskeletal reorganization [77], peak intracellular calcium response in osteoblasts [76], reduced adhesion of β3Y747A mutant platelets to fibrinogen [74], onset of cardiac cell apoptosis [78], and regulation of von Willebrand factor (VWF) production in human umbilical vein endothelial cells (HUVECs) [73]. These literature values correspond to study-specific experimental conditions and biological endpoints and should not be interpreted as directly equivalent or universal WSS limits.

Previous studies have reported that different cell types exhibit specific physiological responses to distinct ranges of WSS, which can vary widely depending on the cell type and the duration of exposure [32]. While some cells tolerate wide ranges of WSS, such as endothelial cells, stem cells, fibroblasts, and bacteria, ranging between 0 and 6 Pa, cancer cells in continuous flow exhibit tolerable ranges varying between 25 and 300 mPa for fibrosarcoma, adenocarcinoma, breast, and ovarian cancer [32]. In these studies, specific WSS conditions have been associated with functional responses such as changes in migration direction, cytoskeletal reorganization, modulation of adhesion, or activation of mechanosensitive signaling pathways [72,73,74,75,76,77,78,79]. The WSS values obtained in the present simulations are compared with the literature-reported values only to provide a mechanobiological context for the simulated hydrodynamic conditions and should not be interpreted as evidence of equivalent cellular responses or as direct biological validation of the model. This comparative analysis is presented in Figure 9, in which the WSS values obtained for the cell-sized probe structures are contextualized against WSS values and ranges previously associated with specific cellular responses in the literature. Because the probes are rigid, wall-attached geometric surrogates, the calculated WSS represents the local hydrodynamic loading on the probe surface and is not equivalent to the total mechanical loading experienced by a living, deformable, and adherent cell.

Accordingly, the geometric WSS calculated on the rigid probe surface should be distinguished from cell-surface traction, pressure-driven interstitial flow, and the total mechanical loading experienced by living cells, which may additionally involve deformation, adhesion, extracellular matrix interactions, and other mechanical contributions. Furthermore, the present wall-attached hemispherical geometry differs from a freely suspended spherical particle, for which hydrodynamic force and torque depend on particle motion and the surrounding flow field; therefore, these models should not be considered mechanically equivalent [80].

The simulated WSS values and selected literature-reported WSS values or ranges from experimental studies are summarized in Table 1 to provide a general mechanical reference for the simulated conditions. The table organizes the reference cell types, the literature-reported WSS values, associated biological responses, and simulated conditions presenting comparable WSS magnitudes, facilitating contextual comparison and interpretation of the results.

The simulated values obtained for the generic probe cells overlapped with WSS magnitudes reported in the literature for a variety of specific experimental conditions, ranging from 2D, 3D, and ex vivo platforms to variations in biological inputs, hydraulic resistance, ECM porosity, viscosity, medium density, and inlet flow configuration. Nevertheless, a precise reproduction of the biological responses cited in the referenced articles depends on incorporating the specific medium viscosity, the viscoelastic properties of the cell under study, and the full geometric description of the experimental setup, including its extracellular matrix.

Cross-study comparisons should be considered approximate and intended solely for mechanical contextualization, as biological responses to WSS depend on cell type, exposure duration, flow waveform, substrate or extracellular matrix, medium composition, and measurement methodology.

Accordingly, the literature-based WSS comparison presented in Figure 9 and Table 1 should be interpreted only as a contextual reference derived from previously published experimental studies. These literature-reported values are study-specific and should not be considered universal mechanobiological thresholds or direct predictors of cellular responses in the present device.

The WSS values for the 0.5 µL/min flow rate did not reach WSS ranges reported in the literature for any process found in the investigated articles. While the 1 µL/min flow rate generated WSS values within a range reported in an experimental study involving alterations in the migration direction of osteoblasts positioned in the cell 3 region for S2 and S3 (50 mPa), these conditions provide a mechanical range that may be considered for future experimental investigation of osteoblast activity (70 mPa) [75].

The 5 µL/min flow rate resulted in WSS values exceeding the range reported in an experimental study investigating changes in fibroblast migration direction under the experimental conditions reported in the literature (50 mPa) for cells 1 and 2 in S1 [79]. providing a potential range for future experimental evaluation. The WSS in the same cells 1 and 2 did not show relevant values for S2. However, cell 3 presented WSS values within a range reported in the literature from experiments investigating endothelial cell elongation, as well as the polarization and reorganization of the cytoskeleton of renal cells (100 mPa), as described in other simulation studies [72,77]. This WSS range also overlaps with values reported in experimental studies investigating impaired adhesion of mutant β3Y747A platelets (160 mPa) [74] and apoptosis of cardiac cells (240 mPa) [78], which was also comparable in magnitude to the WSS condition reported in an experimental study investigating VWF production in HUVECs (300 mPa) [73]. For S3, cells 1 and 2 also presented values higher than those for the change in fibroblast migration direction (50 mPa) [79], while cell 3 exhibited the same conditions highlighted in S2, with a higher WSS value, overlapping with a WSS value reported in an experimental study investigating Ca^2+^ responses in osteoblasts [76].

Finally, the absence of an ECM in the model represents a limitation of the present study. The incorporation of a 3D matrix alters the porosity and hydraulic resistance of the system, redistributing the flow and locally attenuating WSS [81]. In addition, the ECM acts as a mechanical filter that redistributes forces and directly influences cellular migration mechanisms [82]. Thus, although the current model provides a quantitative physical characterization of WSS within the device, future studies integrating 3D matrices and experimental validation will be essential to further elucidate the mechanobiological interpretation of the results and to expand the applicability of the device for cell migration studies.

Taken together, these results show that a single global flow condition does not necessarily correspond to a uniform mechanical stimulus at the cellular level. Comparison with the literature-reported WSS values and ranges provides a general mechanical context for the conditions generated in the present simulations, identifying regions whose WSS magnitudes overlap with ranges investigated in previous cellular studies. These comparisons provide a mechanobiological context for the simulated conditions but do not imply that the corresponding biological responses would necessarily occur in the present device. These findings highlight the importance of device geometry and flow configuration in controlling the cellular mechanical microenvironment.

Analysis of the operational scenarios of a three-dimensional microfluidic device designed to support cell migration studies demonstrated that the mechanical conditions acting on the simulated cell-sized probe structures are determined by the combination of applied flow rate, device geometry, flow inlet/outlet configuration, and the spatial distribution of the probe structures within the platform. Velocity, pressure, and WSS represent distinct hydrodynamic quantities and should not be interpreted as interchangeable indicators of mechanical stimulation. Velocity characterizes fluid transport and pressure represents the normal mechanical component of the flow field and its gradients, whereas WSS represents the tangential viscous traction acting at the fluid–solid interface. Therefore, their simultaneous increase with flow rate reflects the hydrodynamic response of the system rather than an equivalent increase in a single mechanobiological stimulus.

Under conditions S1 and S3, a greater possibility of mechanical interference during future cell-based assays was observed, although through distinct mechanisms. In S1, Cells 1 and 2 were more directly influenced by the main flow, while Cell 3 remained practically without mechanical stimulus due to the absence of effective flow in that region. This pattern indicates the formation of an internal mechanical gradient, in which different cell regions are exposed to distinct levels of WSS. In S3, the simultaneous influx of flow into more channels increased the exposure of Cells 1, 2, and 3 to WSS, amplifying the influence of the mechanical stimulus on the platform. Therefore, depending on the flow rate used, S1 and S3 can make the interpretation of cell migration assays more difficult, since cellular processes such as adhesion, cytoskeleton organization, polarization, and mechanosensitive response have been reported to depend on the mechanical conditions imposed by experimental systems [64,83,84].

In the S2 configuration, the redistribution of flow to the lateral channels reduced WSS in Cells 1 and 2, while increasing the mechanical exposure of Cell 3. For cell migration studies specifically involving the central and intermediate channels, S2 may provide a comparatively lower-WSS mechanical environment while maintaining continuous perfusion in these regions. However, this advantage is region-specific and should not be interpreted as indicating greater mechanical homogeneity throughout the entire device or as evidence of improved cell migration. The suitability of S2 from a mechanical perspective therefore depends on the region of interest and the applied flow rate and should be interpreted within the scope of the present computational analysis. To provide a representative quantitative comparison among the configurations, the highest simulated flow rate (5 µL/min) was selected, as it more clearly highlights the spatial differences in WSS. At this flow rate, the mean probe-surface WSS values in S2 were 6.23 and 7.00 mPa for Cells 1 and 2, respectively, compared with 26.39 and 20.56 mPa in S1 and 13.31 and 2.46 mPa in S3 (Supplementary Tables S4–S6). In contrast, Cell 3 in S2 exhibited a substantially higher mean probe-surface WSS of 125.56 mPa. This mean probe-surface WSS should be distinguished from the WSS evaluated at the probe apex, as these values represent different spatial metrics and should not be interpreted interchangeably. This finding further demonstrates that the lower WSS exposure associated with S2 is region-specific. Thus, the outermost channel may represent a preferential pathway for perfusion and system maintenance within the simulated configuration, resulting in comparatively lower flow-induced mechanical exposure in specific regions of the simulated device. This interpretation is consistent with studies showing that interstitial flow and mechanical stresses can direct or modulate cell migration in 3D matrices [38,41].

Taken together, the results indicate that S3 promoted the greatest sensitivity of the WSS to increased flow in the evaluated cell regions, as demonstrated by the larger slope of the linear regressions presented in Figure 8. Between S1 and S2, the configuration exhibiting lower WSS depends on the cell culture region: the lateral channel is less influenced by the WSS in S1, while the central channels show less exposure to the WSS in S2. Thus, for cell migration studies involving the central and intermediate channels, S2 may be more favorable with respect to WSS exposure alone, as it allows flow passage with comparatively lower WSS exposure in these regions under the simulated conditions. These findings reinforce the idea that the choice of flow rate, operational configuration, and cultivation region should be made in an integrated manner when defining the mechanical conditions for future cell migration experiments.

It is important to emphasize that the analyses performed considered cell-sized hemispherical probe structures positioned at the center of the channels and at the base of the device. Although this approach enables direct comparisons between different regions of the chip, higher WSS values may occur near channel walls, and cell-sized structures positioned at different vertical locations may experience distinct mechanical stimuli (Figure S3) [85]. The simulations also assume steady laminar flow of a Newtonian, water-like fluid, no-slip conditions at the solid boundaries, rigid channel walls, and idealized micropore geometries. These assumptions simplify the experimental conditions, since variations in fluid properties, pore dimensions, and channel geometry may modify the local hydrodynamic field. In addition, deformation or collapse of PDMS microchannels may alter the effective channel dimensions and consequently the local flow distribution [54,55]. The present computational framework represents an idealized mechanical environment and does not account for device-specific factors such as material deformation, fabrication tolerances, surface roughness, or uncertainty in cell positioning. These effects are highly dependent on the specific experimental platform and should be considered in future device-specific experimental investigations. These considerations highlight the inherent complexity of the mechanical microenvironment in 3D microfluidic devices and should be taken into account when designing in vitro experiments.

## 5. Limitations

This study presents some limitations that should be considered when interpreting the results. First, the present study was entirely based on numerical modeling, and no experiments with biological cells were performed. The scope of the present study was therefore restricted to the computational characterization of the mechanical microenvironment, while experimental validation of the predicted flow conditions was not addressed and should be considered in future studies. In this context, the cellular responses discussed in relation to the simulated WSS ranges were inferred from previously published studies and were not experimentally validated in the present device. Accordingly, these comparisons should be interpreted as providing a mechanobiological context for the simulated mechanical conditions rather than as predictions of actual cell behavior. Another limitation is the use of water as a simplified reference fluid. Cell-culture, serum-containing, or matrix-containing media may exhibit different viscosities, which could modify the magnitude of the calculated WSS. In addition, the cell-sized hemispherical probe structures were represented as rigid geometries, and variations in cell size, deformability, and viscoelastic properties were not considered. These characteristics can directly influence fluid–structure interactions, thereby altering local stresses and the effective mechanobiological stimulus depending on the specific cell type represented in the model [86,87]. Since the mechanical response of cells depends on these characteristics, the incorporation of deformable cell models of different dimensions could provide estimates closer to the biological and physiological conditions observed experimentally. Furthermore, the present model was not designed to reproduce the full complexity of a physiological tissue or a multicellular organ-on-a-chip system. Therefore, cell–cell interactions, cell–extracellular matrix interactions, extracellular matrix remodeling, biochemical signaling, and other collective biological processes associated with cell migration were not considered in the present analysis. The focus of this work was to quantitatively characterize the local mechanical microenvironment generated by the platform. Accordingly, the present results should be interpreted within this mechanical framework rather than as predictions of cell migration behavior. Moreover, the analysis was conducted using a single microfluidic device geometry. Although this design represents architecture widely used in cell migration studies, different geometries, channel dimensions, micropore configurations, and perfusion strategies may modify the local distribution of velocity, pressure, and WSS. Therefore, the results obtained should be interpreted within the context of the investigated platform.

Future studies could expand this approach to different microfluidic architectures and more complex cell models, including deformable cells, extracellular matrix representations, multicellular systems, and coupled biomechanical–biological models, contributing to a more accurate representation of the mechanobiological conditions found in physiologically relevant microenvironments.

## 6. Conclusions

This study demonstrated that varying the flow rate and inlet and outlet configurations in a three-dimensional microfluidic device designed for cell migration studies modifies the distribution of velocity, pressure, flow, and WSS, generating distinct mechanical microenvironments within the same platform. Although these parameters increased proportionally with the applied flow rate, their spatial distribution was strongly influenced by the device geometry, the operational configuration, and the location of the cell-sized probe structures within the microfluidic platform.

The local analysis demonstrated that identical global perfusion conditions can generate substantially different WSS magnitudes depending on the position within the device. These findings emphasize the importance of quantitatively characterizing the mechanical microenvironment before performing biological assays, since local variations in WSS may influence the interpretation of experimental results. Comparison with WSS ranges reported in the literature further showed that some of the simulated values overlap with ranges previously associated with different mechanobiological responses in experimental studies. However, these comparisons are literature-based and cell-type-specific and therefore should not be interpreted as direct predictions of cellular responses under the present conditions.

Among the conditions evaluated, S2 maintained continuous perfusion while producing comparatively lower WSS values in the central and intermediate channels. This characterization is restricted to the specific regions and mechanical criteria evaluated and should not be interpreted as indicating greater mechanical homogeneity throughout the entire device or as evidence that S2 improves cell migration. Thus, the proposed in silico computational framework provides quantitative criteria for selecting flow rate, operational configuration, and culture region according to the targeted mechanical conditions.

Overall, this work establishes a quantitative mechanical framework that supports the rational design, optimization, and interpretation of three-dimensional microfluidic platforms for mechanobiology and future cell migration studies. Rather than predicting cellular behavior, the proposed approach provides a robust characterization of the device-generated mechanical microenvironment, serving as a foundation for subsequent experimental investigations and the development of more reliable organ-on-a-chip and microfluidic systems. Experimental validation will be necessary to determine whether the simulated differences in WSS translate into measurable changes in cellular behavior and migration within the evaluated microfluidic configurations.

## Figures and Tables

**Table 1:** Comparison of simulated WSS values with literature-reported WSS values and ranges associated with cellular responses.

Cell Type	WSS Reference (mPa)	Reported Biological Response	Simulated Conditions Within the Reported WSS Range
Fibroblasts [79]	50	Change in migration direction	Probe 3 in S2 and S3 at 1 µL/min; Probe 1 in S1 and Probes 1 and 2 in S3 at 5 µL/min
Osteoblasts [75]	70	Increased osteoblast activity	Probe 3 in S2 and S3 at 1 µL/min and higher-WSS conditions at 5 µL/min
Endothelial cells [72]	100	Cell elongation	Probe 3 in S2 and S3 at 5 µL/min
Renal cells [77]	100	Cell polarization and cytoskeletal reorganization	Probe 3 in S2 and S3 at 5 µL/min
β3Y747A platelets [74]	160	Reduced adhesion to fibrinogen	Higher WSS values observed for Probe 3 at 5 µL/min
Cardiac cells [78]	240	Onset of apoptosis	Probe 3 in S2 and S3 at 5 µL/min
HUVECs [73]	300	VWF production	Highest WSS conditions, particularly Probe 3 at 5 µL/min
Osteoblasts [76]	30–300	Peak intracellular Ca^2+^ production	Probe 3 in S3 at 5 µL/min

Abbreviations: VWF: von Willebrand factor; WSS: wall shear stress.

## Data Availability

The numerical data, STL geometry files, and NASTRAN mesh files supporting this study will be made available through the Open Science Framework (OSF) at: DOI: 10.17605/OSF.IO/Q359A. Solver settings and boundary conditions are described in the manuscript.

## Supplementary Materials

The following supporting information can be downloaded at https://www.mdpi.com/article/10.3390/biomedicines14091879/s1, Table S1: Mesh-independence analysis, grid-convergence index (GCI), and mesh-quality analysis based on the COMSOL mesh-quality metric for situation 1; Table S2: Mesh-independence analysis, grid-convergence index (GCI), and mesh-quality analysis based on the COMSOL mesh-quality metric for situation 2; Table S3: Mesh-independence analysis, grid-convergence index (GCI), and mesh-quality analysis based on the COMSOL mesh-quality metric for situation 3; Figure S1: WSS distribution using the complete color scale for each simulated condition. Expanded-scale visualization of the WSS distribution for scenarios S1, S2, and S3; Table S4: Quantitative summary of wall shear stress (WSS) values on each probe surface for situation 1. Minimum, maximum, mean, and 95th-percentile WSS values are reported for each probe and flow condition; Table S5: Quantitative summary of wall shear stress (WSS) values on each probe surface for situation 2. Minimum, maximum, mean, and 95th-percentile WSS values are reported for each probe and flow condition; Table S6: Quantitative summary of wall shear stress (WSS) values on each probe surface for situation 3. Minimum, maximum, mean, and 95th-percentile WSS values are reported for each probe and flow condition; Table S7: Linear regression parameters for WSS as a function of applied flow for situation 1; Table S8: Linear regression parameters for WSS as a function of applied flow for situation 2; Table S9: Linear regression parameters for WSS as a function of applied flow for situation 3; Figure S2: Mesh-independence assessment of WSS in the micropore regions; Figure S3: Wall shear stress (WSS) distribution on cells positioned in different regions of the microfluidic device. Cells 3a–3i are located in the side channel, Cells 2a–2i in the intermediate channel, and Cells 1a–1o in the central channel. Two stress scales (0–20 mPa and 0–100 mPa) are presented for better visualization of the different WSS levels to which the cells are subjected.
