# Supplementary material for: In Silico Evaluation of Mechanobiological Parameters Under Variable Flow in Three-Dimensional Microfluidic Platforms Supporting Future Cell Migration Studies
Source: Biomedicines. 2026 Aug 23;14(9):1879. doi: 10.3390/biomedicines14091879 (PMC13604881; doi:10.3390/biomedicines14091879)

Supplementary Material

Supplementary Table S1: Mesh-independence analysis, grid-convergence index (GCI), and mesh-quality analysis based on the COMSOL mesh-quality metric for situation 1.

Parameter	Extremely fine	Finer	Normal
Mesh vertices	8088468	2898174	495699
Number of tetrahedras	34461386	10384323	1352614
Number of pyramids	48980	40748	22296
Number of prisms	3728888	1830840	410044
Number of triangles	1906396	948192	220226
Number of quads	128	96	48
Edge elements	63776	46443	19207
Vertex elements	2065	2065	2065
Total of elements	38239254	12255911	1784954
Minimum element quality (Skewness)	0.09094	0.09029	0.02121
Average element quality (Skewness)	0.6932	0.6871	0.6608
Element volume ratio	5.415e-9	1.772e-9	4.101e-10
Mesh volume (mm^3)	3.576	3.576	3.575
h (mm)	0.0045	0.0066	0.0126
Refinement ratio	--	1.461	1.9
r		1.3	
WSS cell apex 1 for 5 µL/min (Pa)	0.06228	0.06053	0.02351
WSS cell apex 2 for 5 µL/min (Pa)	0.04771	0.04709	0.01995
WSS cell apex 3 for 5 µL/min (Pa)	0.00038	0.00037	0.00009
Δ cell apex 1 (%)	--	2.81	61.16
Δ cell apex 2 (%)	--	1.30	57.63
Δ cell apex 3 (%)	--	2.63	75.68
GCI cell apex 1 (%)	--	0.77	4.40
GCI cell apex 2 (%)	--	0.21	1.86
GCI cell apex 3 (%)	--	0.58	3.98

Supplementary Table S2: Mesh-independence analysis, grid-convergence index (GCI), and mesh-quality analysis based on the COMSOL mesh-quality metric for situation 2.

Parameter	Extremely fine	Finer	Normal
Mesh vertices	8086373	2896502	495301
Number of tetrahedras	34458789	10378330	1351795
Number of pyramids	48980	40748	22296
Number of prisms	3725530	1829406	409324
Number of triangles	1906274	948250	220232
Number of quads	590	386	232
Edge elements	63776	46443	19207
Vertex elements	2065	2065	2065
Total of elements	38233299	12248484	1783415
Minimum element quality (Skewness)	0.08338	0.09008	0.0221

Average element quality (Skewness)	0.6931	0.6856	0.6604
Element volume ratio	5.546e-9	1.479e-9	7.695e-10
Mesh volume (mm^3)	3.576	3.576	3.575
h (mm)	0.0045	0.0066	0.0126
Refinement ratio	--	1.461	1.901
r		1.3	
WSS cell apex 1 for 5 μL/min (Pa)	0.01603	0.01563	0.00594
WSS cell apex 2 for 5 μL/min (Pa)	0.01776	0.01759	0.00697
WSS cell apex 3 for 5 μL/min (Pa)	0.32105	0.31470	0.08185
Δ cell apex 1 (%)	--	2.50	62.00
Δ cell apex 2 (%)	--	0.96	60.38
Δ cell apex 3 (%)	--	1.98	73.99
GCI cell apex 1 (%)	--	0.61	3.83
GCI cell apex 2 (%)	--	0.12	1.33
GCI cell apex 3 (%)	--	0.36	2.89

Supplementary Table S3: Mesh-independence analysis, grid-convergence index (GCI), and mesh-quality analysis based on the COMSOL mesh-quality metric for situation 3.

Parameter	Extremely fine	Finer	Normal
Mesh vertices	8086749	2898190	495580
Number of tetrahedras	34461241	10388726	1353084
Number of pyramids	48980	40748	22302
Number of prisms	3725306	1829254	409370
Number of triangles	1906342	948256	220300
Number of quads	654	434	256
Edge elements	63776	46444	19207
Vertex elements	2065	2065	2065
Total of elements	38235527	12258728	1784756
Minimum element quality (Skewness)	0.09037	0.09347	0.00074
Average element quality (Skewness)	0.6917	0.6872	0.6604
Element volume ratio	6.942e-9	2.544e-9	8.547e-10
Mesh volume (mm^3)	3.576	3.576	3.575
h (mm)	0.0045	0.0066	0.0126
Refinement ratio	--	1.461	1.901
r		1.3	
WSS cell apex 1 for 5 μL/min (Pa)	0.06800	0.06656	0.02442
WSS cell apex 2 for 5 μL/min (Pa)	0.06262	0.06101	0.01910
WSS cell apex 3 for 5 μL/min (Pa)	0.34046	0.33290	0.10567
Δ cell apex 1 (%)	--	2.12	63.61
Δ cell apex 2 (%)	--	2.57	68.69
Δ cell apex 3 (%)	--	2.22	68.26
GCI cell apex 1 (%)	--	0.45	3.17
GCI cell apex 2 (%)	--	0.60	3.91
GCI cell apex 3 (%)	--	0.47	3.32

Supplementary Figure S1: WSS distribution using the complete color scale for each simulated condition. Expanded-scale visualization of the WSS distribution for scenarios S1, S2, and S3.

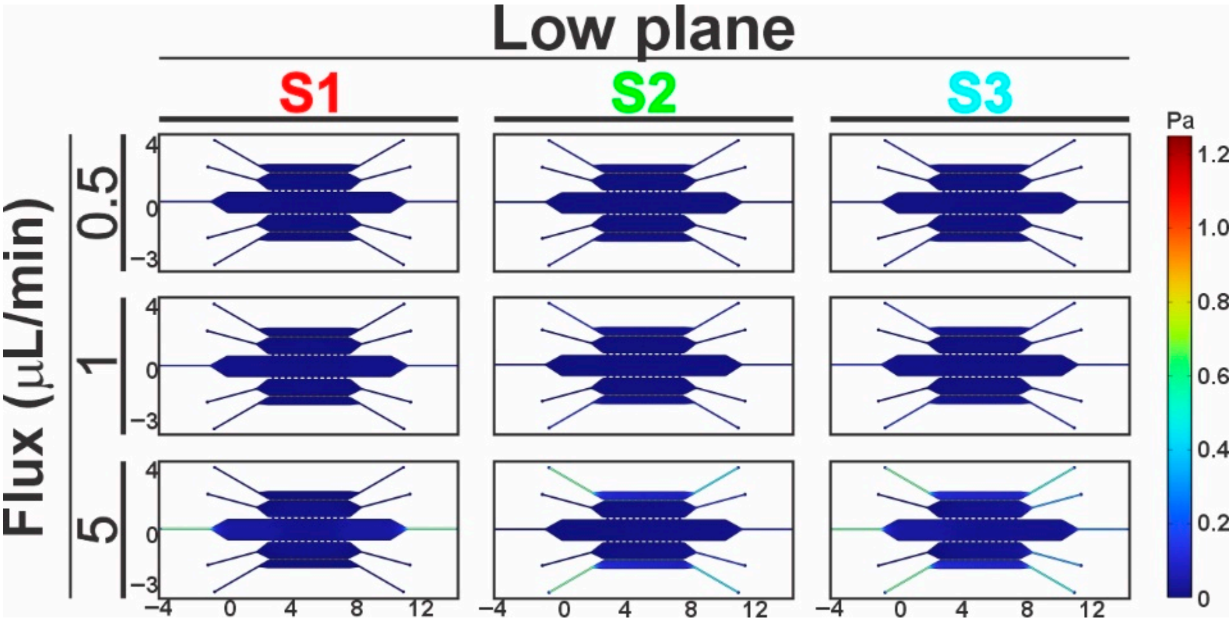

Supplementary Table S4: Quantitative summary of wall shear stress (WSS) values on each probe surface for situation 1. Minimum, maximum, mean, and 95th-percentile WSS values are reported for each probe and flow condition.

Flow (μL/min)	Cell	Mean (Pa)	Minimum (Pa)	Median (Pa)	Maximum (Pa)	P95 (Pa)
	1	0.00264	2.11383E-5	0.00231	0.00616	0.00586
0.5	2	0.00206	2.25785E-5	0.00181	0.0048	0.00457
	3	1.62615E-5	1.51239E-7	1.43376E-5	3.76749E-5	3.60634E-5
	1	0.00528	4.23036E-5	0.00461	0.01231	0.01173
1	2	0.00411	4.52024E-5	0.00362	0.0096	0.00914
	3	3.25254E-5	3.02518E-7	2.8677E-5	7.53561E-5	7.2133E-5
	1	0.02639	2.1152E-4	0.02307	0.06157	0.05863
5	2	0.02056	2.25989E-4	0.01811	0.04798	0.04571
	3	1.62628E-4	1.51262E-6	1.43386E-4	3.76782E-4	3.60666E-4

Supplementary Table S5: Quantitative summary of wall shear stress (WSS) values on each probe surface for situation 2. Minimum, maximum, mean, and 95th-percentile WSS values are reported for each probe and flow condition.

Flow (μL/min)	Cell	Mean (Pa)	Minimum (Pa)	Median (Pa)	Maximum (Pa)	P95 (Pa)
0.5	1	6.2244E-4	1.86069E-5	5.06942E-4	0.00159	0.00156
	2	6.99647E-4	2.2216E-5	5.34566E-4	0.00179	0.00174
	3	0.01256	3.94143E-4	0.0103	0.0318	0.03134
1	1	0.00124	3.72146E-5	0.00101	0.00317	0.00313
	2	0.0014	4.44329E-5	0.00107	0.00358	0.00348
	3	0.02511	7.8834E-4	0.02061	0.06359	0.06268
5	1	0.00623	1.86097E-4	0.00507	0.01587	0.01563
	2	0.007	2.22171E-4	0.00535	0.01791	0.01738
	3	0.12556	0.00394	0.10298	0.31785	0.31328

Supplementary Table S6: Quantitative summary of wall shear stress (WSS) values on each probe surface for situation 3. Minimum, maximum, mean, and 95th-percentile WSS values are reported for each probe and flow condition.

Flow (μL/min)	Cell	Mean (Pa)	Minimum (Pa)	Median (Pa)	Maximum (Pa)	P95 (Pa)
	1	0.13306	0.00391	0.10996	0.33642	0.33084
0.5	2	0.02458	7.90058E-4	0.02035	0.06208	0.06123
	3	0.0265	7.87237E-4	0.02175	0.06699	0.06588
	1	0.02661	7.82059E-4	0.02199	0.06731	0.06619
1	2	0.00492	1.58032E-4	0.00407	0.01242	0.01225
	3	0.0053	1.57419E-4	0.00435	0.0134	0.01318
	1	0.01331	3.91079E-4	0.011	0.03365	0.0331
5	2	0.00246	7.90126E-5	0.00203	0.00621	0.00612
	3	0.00265	7.87062E-5	0.00217	0.0067	0.00659

Supplementary Table S7: Linear regression parameters for WSS as a function of applied flow for situation 1.

Situation 1	Cell 1	Cell 2	Cell 3
Flow (μL/min)	WSS (Pa)		
0.5	0.00609	0.00475	0.00003
1.0	0.01218	0.00950	0.00008
5	0.06088	0.04752	0.00038
**Situation 1**	**Cell 1**	**Cell 2**	**Cell 3**
**Regression parameters**			
Intercept	-4.68214e-7	-1.20629e-6	-5.08496e-10
Intercept standard error	4.11463e-7	5.95302e-7	3.73155e-10
Slope	0.01218	0.0095	7.50197e-5
Slope standard error	1.391e-7	2.01249e-7	1.26149e-10
Residual sum of squares	2.35411e-13	4.92762e-13	1.93616e-19

Supplementary Table S8: Linear regression parameters for WSS as a function of applied flow for situation 2.

Situation 2	Cell 1	Cell 2	Cell 3
Flow (μL/min)	WSS (Pa)		
0.5	0.00156	0.00176	0.03147
1.0	0.00313	0.00352	0.06294
5	0.01563	0.01759	0.31470
**Situation 2**	**Cell 1**	**Cell 2**	**Cell 3**
**Regression parameters**			
Intercept	2.07083e-6	-3.04367e-7	-2.84756e-7
Intercept standard error	1.20589e-6	9.34063e-8	8.01829e-8
Slope	0.06294	0.00352	0.00313
Slope standard error	4.07664e-7	3.15771e-8	2.71068e-8
Residual sum of squares	2.02198e-12	1.21315e-14	8.93978e-15

Supplementary Table S9: Linear regression parameters for WSS as a function of applied flow for situation 3.

Situation 3	Cell 1	Cell 2	Cell 3
Flow (μL/min)	WSS (Pa)		
0.5	0.00666	0.00610	0.03329
1.0	0.01331	0.01220	0.06658
5	0.06656	0.06101	0.33290
**Situation 3**	**Cell 1**	**Cell 2**	**Cell 3**
**Regression parameters**			
Intercept	-6.74168e-7	-3.10739e-7	3.10585e-7
Intercept standard error	9.77311e-9	5.44135e-8	5.55737e-7

Slope	0.01331	0.0122	0.06658
Slope standard error	3.30391e-9	1.83951e-8	1.87873e-7
Residual sum of squares	1.3281e-16	4.11697e-15	4.29439e-13

Supplementary Figure S2: Mesh-independence assessment of WSS in the micropore regions.

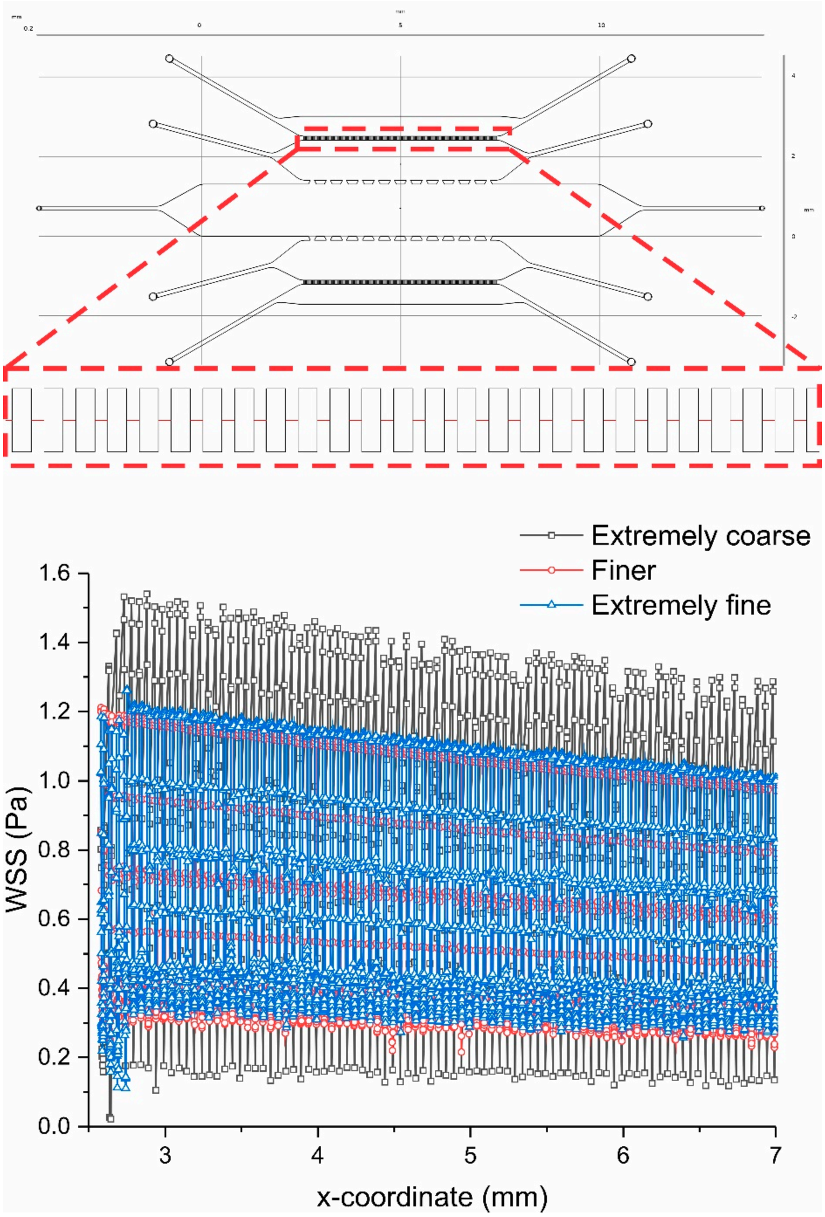

Supplementary Figure S3: Wall shear stress (WSS) distribution on cells positioned in different regions of the micro-fluidic device. Cells 3a–3i are located in the side channel, Cells 2a–2i in the intermediate channel, and Cells 1a–1o in the central channel. Two stress scales (0–20 mPa and 0–100 mPa) are presented for better visualization of the different WSS levels to which the cells are subjected.

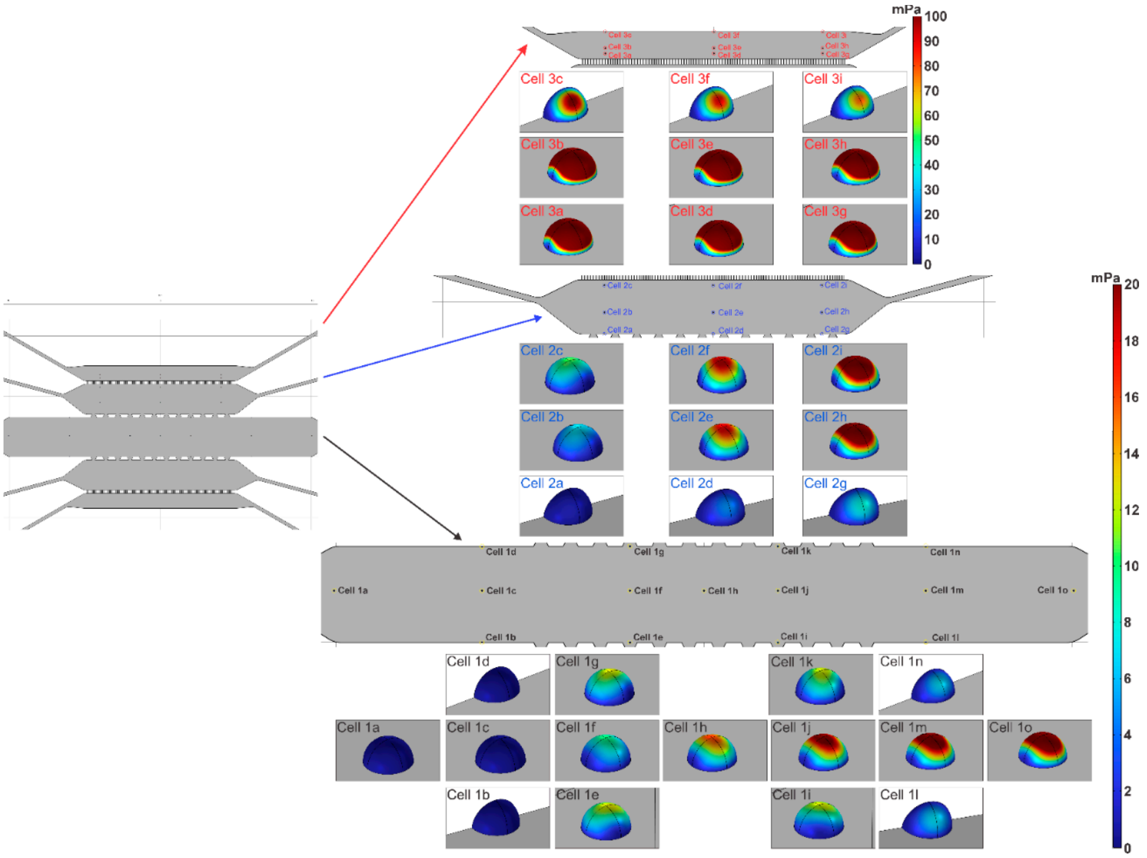

Supplement: Supplementary file 1 [file biomedicines-14-01879-s001.zip › biomedicines-4469809-supplementary.pdf]
